# Whole-Genome Comparisons of Ergot Fungi Reveals the Divergence and Evolution of Species within the Genus *Claviceps* Are the Result of Varying Mechanisms Driving Genome Evolution and Host Range Expansion

**DOI:** 10.1093/gbe/evaa267

**Published:** 2021-01-29

**Authors:** Stephen A Wyka, Stephen J Mondo, Miao Liu, Jeremy Dettman, Vamsi Nalam, Kirk D Broders

**Affiliations:** 1 Department of Agricultural Biology, Colorado State University, Fort Collins, Colorado, USA; 2 U.S. Department of Energy Joint Genome Institute, Berkeley, California, USA; 3 Ottawa Research and Development Centre, Agriculture and Agri-Food Canada, Ottawa, Ontario, Canada; 4 Smithsonian Tropical Research Institute, Panamá, República de Panamá

**Keywords:** adaptive evolution, gene cluster expansion, fungal plant pathogens, RIP

## Abstract

The genus *Claviceps* has been known for centuries as an economically important fungal genus for pharmacology and agricultural research. Only recently have researchers begun to unravel the evolutionary history of the genus, with origins in South America and classification of four distinct sections through ecological, morphological, and metabolic features (*Claviceps* sects. *Citrinae*, *Paspalorum*, *Pusillae*, and *Claviceps*). The first three sections are additionally characterized by narrow host range, whereas section *Claviceps* is considered evolutionarily more successful and adaptable as it has the largest host range and biogeographical distribution. However, the reasons for this success and adaptability remain unclear. Our study elucidates factors influencing adaptability by sequencing and annotating 50 *Claviceps* genomes, representing 21 species, for a comprehensive comparison of genome architecture and plasticity in relation to host range potential. Our results show the trajectory from specialized genomes (sects. *Citrinae* and *Paspalorum*) toward adaptive genomes (sects. *Pusillae* and *Claviceps*) through colocalization of transposable elements around predicted effectors and a putative loss of repeat-induced point mutation resulting in unconstrained tandem gene duplication coinciding with increased host range potential and speciation. Alterations of genomic architecture and plasticity can substantially influence and shape the evolutionary trajectory of fungal pathogens and their adaptability. Furthermore, our study provides a large increase in available genomic resources to propel future studies of *Claviceps* in pharmacology and agricultural research, as well as, research into deeper understanding of the evolution of adaptable plant pathogens.

**Significance**

Lack of genomic data for the *Claviceps* genus has hampered the ability to identify factors influencing the adaptation of *Claviceps* species and mechanisms associated with the broad host range of some species. Our analysis reveals the trajectory from specialized genomes toward adaptive genomes through a variety of genomic mechanisms which coincided with increases in host range potential. These results demonstrate a clear example of how genomic alterations can influence and shape the evolutionary trajectory of fungal pathogens in association with host range.

## Introduction

Fungi, particularly phytopathogenic species, are increasingly being used to gain insight into the evolution of eukaryotic organisms, due to their adaptive nature and unique genome structures (Gladieux et al. 2014; Dong et al. 2015). Adaptation and diversification of fungal species can be mediated by changes in genome architecture and plasticity, such as genome size, transposable element (TE) content, localization of TEs to specific genes, genome compartmentalization, gene duplication rates, recombination rates, and presence/absence polymorphism of virulence factors (Dong et al. 2015; Möller and Stukenbrock 2017). The presence or absence of repeat-induced point (RIP) mutation is also an important mechanism for fungal genome evolution, as RIP works on a genomewide scale to silence TEs and duplicated genes, which can also “leak” onto neighboring genes (Galagan et al. 2003; Galagan and Selker 2004; Raffaele and Kamoun 2012; Urguhart et al. 2018; Möller and Stukenbrock 2017). It is becoming increasingly evident that variations in these factors can be used to classify genomes as a one speed (one compartment), such as the powdery mildew fungi *Blumeria graminis* f.sp. *hordei* and f.sp *tritici*, two speed (two compartments), such as the late blight pathogen *Phytophthora infestans*, or multispeed (multicompartment) such as the multihost pathogen *Fusarium oxysporum* (Dong et al. 2015; Frantzeskakis et al. 2019). These different “speeds” are characterized by their potential adaptability such that one-speed genomes are often considered less adaptable, whereas two-speed and multispeed genomes are often considered more adaptable (Dong et al. 2015; Frantzeskakis et al. 2019; Möller and Stukenbrock 2019).

The ergot fungi of the genus *Claviceps* (Ascomycota, Hypocreales) are biotrophic species that share a specialized ovarian-specific nonsystemic parasitic lifestyle with their grass hosts (Píchová et al. 2018). Infections are fully restricted to individual unpollinated ovaries (Tudzynski and Scheffer 2004), and the fungus actively manages to maintain host cell viability to obtain nutrients from living tissue through a complex cross-talk of genes related to pathogenesis, such as secreted effectors, secondary metabolites, or cytokinin production (Hinsch et al. 2015, 2016; Oeser et al. 2017; Kind, Schurack, et al. 2018; Kind, Hinsch, et al. 2018). Species of *Claviceps* are most notably known for their production of toxic alkaloids and secondary metabolites but are also known for their expansive host range and negative impact on global cereal crop production and livestock farming. These negative effects on human and livestock health are the primary reason *Claviceps* species are referred to as plant pathogens. However, under the light of coevolution with their grass hosts, some *Claviceps* species are considered conditional defensive mutualists with their hosts as they prevent herbivory and can improve host fitness (Raybould et al. 1998; Fisher et al. 2007; Wäli et al. 2013).

The genus *Claviceps* contains 59 species divided into four sections as follows: *Claviceps*, *Pusillae*, *Citrinae*, and *Paspalorum* (Píchová et al. 2018). It was postulated that sections *Citrinae* and *Paspalorum* originated in South America, whereas section *Pusillae* experienced speciation throughout the Eocene, Oligocene, and Miocene as these species encountered newly emergent PACMAD warm-season grasses (subfamilies Panicoideae, Aristidoideae, Chloridoideae, Micrairoideae, Arundinoideae, and Danthonioideae) when an ancestral strain was transferred from South America to Africa (Píchová et al. 2018). In contrast, the crown node of section *Claviceps* is estimated at 20.4 Ma and was followed by a radiation of the section corresponding to a host jump from ancestral sedges (Cyperaceae) to the Bamboo, Oryzoideae, Pooideae (BOP) clade (cool-season grasses; subfamilies Bambusoideae, Oryzoideae [syn: Ehrhartoideae]; Soreng et al. 2017, Pooideae) in North America (Bouchenak-Khelladi et al. 2010; Píchová et al. 2018). Section *Claviceps* has the largest host range with *C. purpurea sensu stricto* (s.s.) having been reported on up to 400 different species in clade BOP (Alderman et al. 2004, Píchová et al. 2018) across six tribes and retains the ability to infect sedges (Cyperaceae) (Jungehülsing and Tudzynski 1997). In contrast, section *Pusillae* is specialized to the tribes Paniceae and Andropogoneae, and sections *Citrinae* and *Paspalorum* only infect members of tribe Paspaleae and tribe Cynodonteae, respectively (Píchová et al. 2018). The shared specialized infection life cycle of the *Claviceps* genus, the drastic differences in host range potential of different species, and geographic distribution represent a unique system to study the evolution and host adaptation of eukaryotic organisms.

Despite their ecological and agriculture importance, little is known about the evolution and genomic architecture of these important fungal species in comparison with other cereal pathogens such as species in the genera *Puccinia* (Cantu et al. 2013; Kiran et al. 2016, 2017), *Zymoseptoria* (Estep et al. 2015; Grandaubert et al. 2015, 2019; Poppe et al. 2015; Testa, Oliver et al. 2015; Wu et al. 2017; Stukenbrock and Dutheil 2018), or *Fusarium* (Kvas et al. 2009; Ma et al. 2010; Rep and Kistler 2010; Watanabe et al. 2011; Sperschneider et al. 2015). Unfortunately, the lack of genome data for the *Claviceps* genus has hampered our ability to complete comparative analyses to identify factors that are influencing the adaptation of *Claviceps* species across the four sections in the genus, and the mechanisms by which species of section *Claviceps* have adapted to such a broad host range, in comparison with the other three sections. Here we present the sequences and annotations of 50 *Claviceps* genomes, representing 19 species, for a comprehensive comparison of the genus to understand evolution within the genus *Claviceps* by characterizing the genomic plasticity and architecture in relation to adaptive host potential. Our analysis reveals the trajectory from specialized one-speed genomes (sects. *Citrinae* and *Paspalorum*) toward adaptive two-speed genomes (sects. *Pusillae* and *Claviceps*) through colocalization of TEs around predicted effectors and a putative loss of RIP resulting in tandem gene duplication coinciding with increased host range potential.

## Materials and Methods

### Sample Acquisition

Field collected samples (Clav) were surfaced sterilized, allowed to grow as mycelia, and individual conidia transferred to make single spore cultures. Thirteen cultures were provided by Dr Miroslav Kolařík from the Culture Collection of Clavicipitaceae (CCC) at Institute of Microbiology, Academy of Sciences of the Czech Republic. Raw Illumina reads for samples (LM28, LM582, LM78, LM81, LM458, LM218, LM454, LM576, and LM583) were downloaded from NCBI SRA database. Raw Illumina reads from an additional 21 LM samples were generated by Dr Liu’s lab (AAFC), sequencing protocol of these 21 samples followed (Wingfield et al. 2018). Summarized information can be found in supplementary table S1, Supplementary Material online.

### Preparation of Genomic DNA

Cultures grown on cellophane PDA plates were used for genomic DNA extraction from lyophilized mycelium following a modified CTAB method (Doyle JJ and Doyle JL 1987; Wingfield et al. 2018) without using the RNase Cocktail Enzyme Mix, only RNase A was used. DNA contamination was checked by running samples on a 1% agarose gel and a NanoDrop One^c^ (Thermo Fishcer Scientific). Twenty samples (7 Clav and 13 CCC) were sent to BGI-Hong Kong HGS Lab for 150-bp paired-end Illumina sequencing on an HiSeq 4000.

### Genome Assembly

Preliminary data showed that raw reads of LM458 were contaminated with bacterial DNA but showed strong species similar to Clav32 and Clav50. To filter out the bacterial DNA sequences, reads of LM458 were mapped against the assembled Clav32 and Clav50 genomes using BBSplit v38.41 (Bushnell 2014). All forward and reverse reads mapped to each of the genomes were concatenated, respectively. Both sets were then interleaved to remove duplicates and used for further analysis. Reads for all 50 samples were checked for quality with FastQC v0.11.5 (Andrews 2010) and trimmed with Trimmomatic v0.36 (Bolger et al. 2014) using the commands (SLIDINGWINDOW: 4:20; MINLEN:36; HEADCROP:10) to remove poor quality data, only paired-end reads were used. To better standardize the comparative analysis, all 50 samples were subject to de novo genome assembly with Shovill v0.9.0 (https://github.com/tseemann/shovill; last accessed May 11, 2020) using SPAdes v3.11.1 (Nurk et al. 2013) with a minimum contig length of 1,000 bp.

The reference genomes of *C. purpurea* strain 20.1 (SAMEA2272775), *C. fusiformis* PRL 1980 (SAMN02981339), and *C. paspali* RRC 1481 (SAMN02981342) were downloaded from NCBI. Proteins for *C. fusiformis* and *C. paspali* were not available on NCBI so they were extracted from GFF3 files provided by Dr Chris Schardl and Dr Neil Moore, University of Kentucky, corresponding to the 2013 annotations (Schardl et al. 2013) available at http://www.endophyte.uky.edu (last accessed March 22, 2020). Reference genomes were standardized for comparative analysis with our 50 annotated genomes, by implementing a protein length cutoff of 50 aa and removal of alternatively spliced proteins in *C. fusiformis* and *C. paspali*, only the longest spliced protein for each locus remained.

### Transposable Elements

TE fragments were identified following procedures for establishment of de novo comprehensive repeat libraries set forth in Coghlan et al. (2018), a brief summary is described below. The following steps were automated through construction of a custom script, TransposableELMT (https://github.com/PlantDr430/TransposableELMT). Each of the 53 *Claviceps* genome were used to create a respective repeat library using RepeatModeler v1.0.8 (Smit and Hubley 2015), TransposonPSI (Hass 2010), and long terminal repear (LTR) LTR_finder v1.07 (Xu and Wang 2007) on default settings. LTR_harvest v1.5.10 (Ellinghaus et al. 2008) was additionally run on default settings, and results were filtered with LTR_digest v1.5.10 (Steinbiss *et al.* 2009) with an HMM search for Pfam domains associated with TEs; only candidates with domain hits were kept. Repeat libraries from these four programs were concatenated with all curated TEs from RepBase (Bao et al. 2015) and redundant sequences were removed using Usearch v11.0.667 (Edgar 2010) with a percent identity cutoff of ≥80%. TEs for each of the nonredundant libraries were classified using RepeatClassifier v1.0.8 (Smit and Hubley 2015). RepeatMasker v4.0.7 (Smit et al. 2015) was then used, on default settings with each assemble genome and its respective repeat library, to soft mask the genomes and identify TE regions. TE content was represented as the proportion of the genome masked by TE regions determined by RepeatMasker, excluding simple and low complexity repeats.

The TE divergences, calculated from RepeatMasker for TEs in all 53 *Claviceps* genomes, were used to plot the divergence landscape using a custom script (https://github.com/PlantDr430/CSU_scripts/blob/master/TE_divergence_landscape.py). The RepeatMasker results were also used with the respective GFF3 file from each genome to calculate the average distance (kb) of each gene to the closest TE fragment on the 5′ and 3′ flanking side. Values were calculated for predicted effectors, noneffector secreted genes, nonsecreted metabolite genes, and all other genes using a custom script (https://github.com/PlantDr430/CSU_scripts/blob/master/TE_closeness.py).

### Genome Annotation

AUGUSTUS v3.2.2 (Mario et al. 2008) was used to create pretrained parameters files using the reference *C. purpurea* strain 20.1, available expressed sequence tag (EST) data from NCBI, and wild-type RNAseq data (SRR4428945) created in Oeser et al. (2017). RNA-seq data was subject to quality check and trimming as above. All three data sets were also used to train parameter files for the ab initio gene model prediction software’s GeneID v1.4.4 (Blanco et al. 2007) and CodingQuarry v2.0 (Testa et al. 2015). GeneID training followed protocols available at http://genome.crg.es/software/geneid/training.html. For CodingQuarry training, RNA transcripts were created de novo using Trinity v2.8.4 (Grabherr et al. 2011) on default settings and EST coordinates were found by mapping the EST data to the reference genome using Minimap2 v2.1 (Li 2018).

Gene models for the 50 genomes were then predicted with GeneID and CodingQuarry using the trained *C. purpruea* parameter files. CodingQuarry prediction was also supplemented with transcript evidence by mapping the available EST and RNA-seq *C. purpurea* data to each genome using Minimap2. BUSCO v3 (Waterhouse et al. 2018) was run on all 50 genomes using the AUGUSTUS *C. purpurea* pretrained parameter files as the reference organism and the Sordariomyceta database. The resulting predicted proteins for each sample were used as training models for ab initio gene prediction using SNAP (Korf 2004) and GlimmerHMM v3.0.1 (Majoros et al. 2004). Last, GeMoMa v1.5.3 (Keilwagen et al. 2016) was used for ab initio gene prediction using the soft-masked genomes and the *C. purpruea* 20.1 reference files.

Funannotate v1.6.0 (Palmer and Stajich 2019) was then used as the primary software for genome annotation. Funannotate additionally uses AUGUSTUS and GeneMark-ES (Ter-Hovhannisyan et al. 2008) for ab initio gene model prediction, Exonerate for transcript and protein evidence alignment, and EVidenceModeler (Hass et al. 2008) for a final weighted consensus. All *C. purpurea* EST and RNAseq data were used as transcript evidence and the Uniport Swiss-Prot database and proteins from several closely related species (*C. purpurea* strain 20.1, *C. fusiformis* PRL1980, *C. paspali* RRC1481, *Fusarium oxysporum f.* sp. *lycopersici* 4287, *Pochonia chlamydosporia* 170, *Ustilago maydis* 521, and *Epichloe festucae* F1) were used as protein evidence. The AUGUSTUS pretrained *C. purpurea* files were used as BUSCO seed species along with the Sordariomyceta database and all five ab initio predictions were passed through the –other_gff flag with weights of 1. The following flags were also used in Funannotate “predict”: –repeats2evm, –optimize_augustus, –soft_mask 1000, –min_protlen 50. BUSCO was used to evaluate annotation completeness using the Dikarya and Sordariomyceta databases (odb9) with –prot on default settings.

### Functional Annotation

Functional analysis was performed using Funannotate “annotate.” The following analyses were also performed on the three reference *Claviceps* genomes. Secondary metabolite clusters were predicted using antiSMASH v5 (Blin et al. 2019) with all features turned on. Functional domain annotations were conducted using eggNOG-mapper v5 (Huerta-Cepas et al. 2017, 2019) on default settings and InterProScan v5 (Jones et al. 2014) with the –goterms flag. Phobius v1.01 (Käll et al. 2007) was used to assist in prediction of secreted proteins. In addition to these analyses Funannotate also performed domain annotations through an HMMer search against the Pfam-A database and dbCAN CAZYmes database, a BlastP search against the MEROPS protease database, and secreted protein predictions with SignalP v4.1 (Nielsen 2017).

For downstream analysis, proteins were classified as secreted proteins if they had signal peptides detected by both Phobius and SignalP and did not possess a transmembrane domain as predicted by Phobius and an additional analysis of TMHMM v2.0 (Krogh et al. 2001). Effector proteins were identified by using EffectorP v2.0 (Sperschneider et al. 2018), with default settings, on the set of secreted proteins for each genome. Transmembrane proteins were identified if both Phobius and TMHMM detected transmembrane domains. Secondary metabolite proteins were identified if they resided within metabolite clusters predicted by antiSMASH. Proteins were classified as having conserved protein domains if they contained any Pfam or IPR domains.

### Gene Family Identification and Classification

OrthoFinder v2.3.3 (Emms and Kelly 2019) was run on default settings using Diamond v0.9.25.126 (Buchfink et al. 2015) to infer groups of orthologous gene clusters (orthogroups) based on protein homology and Markov Cluster Algorithm (MCL) clustering. To more accurately place closely related genes into clusters an additional 78 fungal genomes (supplementary table S3, Supplementary Material online) with emphasis on plant associated fungi of the order Hypocreales were added. To standardize, all 78 additional genomes were subject to a protein length cutoff of 50 amino acids and genomes downloaded from http://www.endophyte.uky.edu had alternatively spliced proteins removed. For downstream analysis, orthogroups pertaining to the 53 *Claviceps* genomes were classified as secreted, predicted effectors, transmembrane, metabolite, and conserved domain orthogroups if ≥50% of the *Claviceps* strains present in a given cluster had at least one protein classified as such.

### Phylogeny and Genome Fluidity

Phylogenetic relationship of all 53 *Claviceps* genomes, with *Fusarium graminearum*, *F. verticillioides*, *Epichloe festucae*, and *E. typhina* as outgroups, was derived from 2,002 single-copy orthologs obtained from our OrthoFinder defined gene clusters (described above). This resulted in a data set of 114,114 amino acids sequences that were concatenated to create a supermatrix and aligned using MAFFT v7.429 (Katoh and Standley 2013) on default settings. Uninformative sites were removed using Gblocks v0.91 (Castresana 2000) on default settings. Due to the large scale of the alignment maximum likelihood reconstruction was performed using FastTree v2.1.11 (Price et al. 2010) using the Whelan and Goldman matrix model of amino acid substitution with the –gamma, –spr 4, –mlacc 2, –slownni, and –slow flag with 1,000 bootstraps. MEGA X (Sudhir et al. 2018) was used for neighbor joining (NJ) reconstruction using the Jones, Taylor, and Thorton matrix model of amino acid substitution with gamma distribution and maximum parsimony (MP) reconstruction using the tree bisection reconstruction (TBR) algorithm with 100 repeated searches. Nodal support for both NJ and MP reconstructions were assessed with 1,000 bootstraps. In addition, an alignment and maximum likelihood (ML) reconstruction was performed on each of the 2,002 protein sequences following the procedure as above (MAFFT, Gblocks, FastTree). A density consensus phylogeny was created from all gene trees using the program DensiTree v2.2.5 (Bouckaert and Heled 2014). PhyBin v0.3-1 (Newton RR and Newton IL 2013) was used to cluster trees from three data sets (1: *Claviceps* genus without outgroups, 2: section *Pusillae* species, and 3: section *Claviceps* species) together to identify frequencies of concordant topologies using the –complete flag with –editdist = 2. To reduce noise, from abundant incomplete lineage sorting in section *Claviceps*, we implemented a –minbranchlen = 0.015 for our *Claviceps* genus data set.

Following methodologies established in Kislyuk et al. (2011) genomic fluidity, which estimates the dissimilarity between genomes by using ratios of the number of unique gene clusters to the total number of gene clusters in pairs of genomes averaged over randomly chosen genome pairs from within a group on *N* genomes, was used to assess gene cluster dissimilarity within the *Claviceps* genus. For a more detailed description refer to Kislyuk *et al.* (2011). Data sets containing gene clusters from representative members of section *Pusillae*, section *Claviceps*, *Clavieps* genus, and all *C. purpurea* strains were extracted from our OrthoFinder defined gene clusters. Additional species- and genus-wide gene cluster data sets from the additional 78 fungal genomes were extracted for comparative purposes. All section- and genus-wide data sets contained one representative isolate from each species to reduce phylogenetic bias. Each extracted data set was used to calculate the genomic fluidity using a custom script (https://github.com/PlantDr430/CSU_scripts/blob/master/pangenome_fluidity.py). The result files for each data set were then used for figure creation and two-sample two-sided *z* test statistics (Kislyuk et al. 2011) using a custom script (https://github.com/PlantDr430/CSU_scripts/blob/master/combine_fluidity.py).

### Gene Density Compartmentalization

A custom script (https://github.com/PlantDr430/CSU_scripts/blob/master/genome_speed_hexbins.py) was used to calculate local gene density measured as 5′ and 3′ flanking distances between neighboring genes (intergenic regions). To statistically determine whether specific gene types had longer intergenic flanking regions than all other genes within the genome we randomly sampled 100 each group of genes (specific gene vs. other genes) 1,000 times for both the 5′ and 3′ flanking distances. Mann–Whitney *U* test was used to test for significance on all 2,000 subsets corrected with Benjamini–Hochberg. Corrected *P* values were averaged per flanking side and then together to get a final *P* value. Genes that appeared on a contig alone were excluded from analysis (supplementary table S4, Supplementary Material online). For graphical representation, genes that were located at the start of each contig (5′ end) were plotted along the *x* axis, whereas genes located at the end of each contig (3′ end) were plotted along the *y* axis.

### RIP and Blast Analyses

For all 53 genomes a self-BlastP v2.9.0+ search was conducted to identify best hit orthologs within each genome with a cutoff *e*-value of 10^−5^ and removal of self-hits. This process was automated using a custom script (https://github.com/PlantDr430/CSU_scripts/blob/master/RIP_blast_analysis.py). We further examined if gene pairs with a pairwise identity of ≥80% were located next to each other and/or separated by five or fewer genes. Fifty-six important *Claviceps* genes (supplementary table S7, Supplementary Material online) including the *rid-1* homolog (Freitag et al. 2002) were used in a BlastP analysis to identify the number of genes present that passed an *e*-value cutoff of 10^−5^, 50% coverage, and 35% identity. Genes that appeared as best hits for multiple query genes were only recorded once for their overall best match. In addition, the web-based tool The RIPper (Van Wyk et al. 2019) was used on default settings (1-kb windows in 500-bp increments) to scan whole genomes for presence of RIP and large RIP affected regions (LRARs).

### Statistical Programs and Plotting

Statistics and figures were generated using Python3 modules SciPy v1.3.1, statsmodel v0.11.0, and Matplotlib v3.1.1. Heatmaps were generated using ComplexHeatmap v2.2.0 in R (Gu 2016).

## Results

### Genome Assembly and Annotation

To provide a comprehensive view of variability across *Claviceps*, we sequenced and annotated 50 genomes (19 *Claviceps* spp.), including *C. citrina* the single species of section *Citrinae*, six species belonging to section *Pusillae*, and 44 genomes (12 species) belonging to section *Claviceps*, of which 23 genomes belong to *C. purpurea* s.s. (table 1 and supplementary table S1, Supplementary Material online). The assemblies and annotations were of comparable quality to the reference strains (table 1). A more detailed representation of the assembly and annotation statistics can be seen in table 1 and supplementary figure S1 and table S2, Supplementary Material online.

**Table 1:** Assembly and Annotations Statistics for the Three Reference *Claviceps* Genomes and the 50 *Claviceps* Genomes Used in This Study

Organism	Strain	Section	Host of Origin		Read Coverage	Genome size (Mb)	Contig (#)	N50	Genomic GC (%)	TE Content (%)	Gene Count	BUSCO Completeness	
			Family/Tribe	Genus/Species								Dikarya (%)	Sordario-myceta (%)
**References**													
*C. purpruea*	20.1	Claviceps	Triticeae	*Secale cereale*	—	32.1	1,442^b^	46,498^b^	51.6	10.9	8,703	95.30	94.70
*C. fusiformis*	PRL1980	Pusillae	Paniceae	*Pennisetum typhoideum*	—	52.3	6,930	19,980	37.3	47.5	9,304	96.70	94.90
*C. paspali*	RRC1481	Paspalorum	Paspaleae	*Paspalum* sp.	—	28.9	2,304	26,898	47.7	17.5	8,400	94.30	93.30
**This study**													
*C. purpruea*	Clav04	Claviceps	Bromeae	*Bromus inermis*	46×	31.8	3,288	21,051	51.7	10.1	8,824	95.50	94.10
*C. purpruea*	Clav26	Claviceps	Triticeae	*Hordeum vulgare*	59×	30.8	1,361	49,697	51.7	9.1	8,737	97.70	96.50
*C. purpruea*	Clav46	Claviceps	Triticeae	*Secale cereale*	58×	30.8	1,409	49,302	51.7	9.7	8,597	98.00	96.60
*C. purpruea*	Clav55	Claviceps	Poeae	*Lolium perenne*	59×	30.7	1,525	44,299	51.8	9.8	8,480	97.10	95.90
*C. purpruea*	LM4	Claviceps	Triticeae	*Tricosecale*	64×	30.6	1,296	47,441	51.8	10.0	8,470	97.00	95.80
*C. purpruea*	LM5	Claviceps	Triticeae	*Hordeum vulgare*	67×	30.5	1,258	51,505	51.8	9.0	8,508	96.90	95.50
*C. purpruea*	LM14	Claviceps	Triticeae	*Hordeum vulgare*	49×	30.6	1,297	49,955	51.8	10.0	8,422	97.40	95.60
*C. purpruea*	LM28	Claviceps	Triticeae	*Triticum aestivum*	49×	30.6	1,343	51,635	51.7	9.6	8,713	97.30	96.10
*C. purpruea*	LM30	Claviceps	Triticeae	*Secale cereale*	64×	30.6	1,224	51,374	51.8	9.4	8,526	97.00	95.50
*C. purpruea*	LM33	Claviceps	Triticeae	*Secale cereale*	45×	30.5	1,398	44,564	51.8	9.2	8,557	96.30	95.50
*C. purpruea*	LM39	Claviceps	Triticeae	*Triticum turgidum* subsp. *durum*	81×	30.5	1,282	48,443	51.8	10.1	8,591	97.10	96.10
*C. purpruea*	LM46	Claviceps	Triticeae	*Triticum turgidum* subsp. *durum*	79×	30.6	1,291	50,932	51.8	9.6	8,455	97.00	95.80
*C. purpruea*	LM60	Claviceps	Poeae	*Avena sativa*	81×	30.6	1,259	47,464	51.7	9.3	8,498	97.00	95.80
*C. purpruea*	LM71	Claviceps	Poeae	*Alopercurus myosuroides*	168×	30.5	1,400	45,114	51.8	9.6	8,472	97.10	95.60
*C. purpruea*	LM207	Claviceps	Triticeae	*Elymus repens*	53×	30.5	1,352	45,388	51.8	9.2	8,475	97.00	95.70
*C. purpruea*	LM223	Claviceps	Bromeae	*Bromus riparius*	74×	30.8	1,297	46,577	51.7	10.5	8,438	97.00	95.70
*C. purpruea*	LM232	Claviceps	Poeae	*Phalaris canariensis*	53×	30.7	1,348	49,571	51.7	9.4	8,512	96.60	95.70
*C. purpruea*	LM233	Claviceps	Poeae	*Phalaris canariensis*	49×	30.6	1,331	50,327	51.8	9.9	8,717	96.70	95.90
*C. purpruea*	LM461	Claviceps	Triticeae	*Elymus repens*	37×	30.5	1,440	44,216	51.8	8.4	8,656	96.60	95.20
*C. purpruea*	LM469	Claviceps	Triticeae	*Triticum aestivum*	75×	30.5	1,257	48,403	51.8	10.0	8,394	97.30	96.00
*C. purpruea*	LM470	Claviceps	Triticeae	*Elymus repens*	26×	30.5	1,797	32,579	51.8	9.0	8,591	96.50	95.30
*C. purpruea*	LM474	Claviceps	Triticeae	*Hordeum vulgare*	64×	30.6	1,354	47,245	51.8	9.4	8,500	96.80	95.70
*C. purpruea*	LM582	Claviceps	Triticeae	*Secale cereale*	89×	30.7	1,600	39,003	51.8	9.6	8,518	97.20	95.40
*C. aff. purpruea*	Clav52	Claviceps	Poeae	*Poa pratensis*	60×	29.6	1,334	48,893	51.8	8.2	8,316	96.80	96.20
*C. quebecensis* ^a^	Clav32	Claviceps	Triticeae	*Hordeum vulgare*	64×	28.7	1,068	58,118	51.6	4.5	8,232	98.00	96.60
*C. quebecensis* ^a^	Clav50	Claviceps	Triticeae	*Elymus* sp.	59×	28.8	1,075	66,795	51.6	6.9	8,046	97.50	96.30
*C. quebecensis* ^a^	LM458	Claviceps	Poeae	*Ammophila* (plant)	78×	28.4	1,166	45,693	51.6	6.1	8,055	97.10	95.80
*C. occidentalis* ^a^	LM77	Claviceps	Poeae	*Phleum pratense*	58×	28.7	1,728	29,222	51.4	6.0	8,162	96.10	94.70
*C. occidentalis* ^a^	LM78	Claviceps	Bromeae	*Bromus inermis*	64×	28.8	1,689	29,608	51.4	6.0	8,231	95.80	94.70
*C. occidentalis* ^a^	LM84	Claviceps	Bromeae	*Bromus inermis*	164×	28.9	1,404	36,685	51.4	6.0	8,221	97.00	95.40
*C. ripicola* ^a^	LM218	Claviceps	Poeae	*Phalaris arundinacea*	146×	31.1	1,072	60,464	51.4	10.3	8,327	96.70	95.70
*C. ripicola* ^a^	LM219	Claviceps	Poeae	*Phalaris arundinacea*	55×	30.8	1,239	55,312	51.4	9.5	8,381	96.80	95.80
*C. ripicola* ^a^	LM220	Claviceps	Poeae	*Phalaris arundinacea*	91×	30.9	1,223	54,100	51.4	9.3	8,449	97.10	95.90
*C. ripicola* ^a^	LM454	Claviceps	Poeae	*Ammophila breviligulata*	156×	31.2	1,508	40,844	51.4	8.4	8,562	97.10	96.10
*C. spartinae*	CCC535	Claviceps	Zoysieae	*Sporobolus anglicus*	60×	29.3	1,456	42,688	51.4	7.1	8,433	97.50	95.90
*C. arundinis*	LM583	Claviceps	Molinieae	*Phragmites australis*	69×	30.6	996	70,672	51.4	9.8	8,235	96.80	95.70
*C. arundinis*	CCC1102	Claviceps	Molinieae	*Phragmites australis*	61×	30.3	896	91,905	51.4	8.3	8,486	97.70	96.50
*C. humidiphila*	LM576	Claviceps	Poeae	*Dactylis* sp.	77×	31.2	1,236	55,717	51.5	9.9	8,440	97.00	95.90
*C. perihumidiphila* ^a^	LM81	Claviceps	Triticeae	*Elymus albicans*	140×	31.2	1,003	67,487	51.5	11.0	8,291	97.10	95.90
*C. cyperi*	CCC1219	Claviceps	Cyperaceae (family)	*Cyperus esculentus*	56×	26.6	1,921	27,113	51.7	8.9	7,673	97.70	95.40
*C. capensis*	CCC1504	Claviceps	Ehrharteae	*Ehrharta villosa*	66×	27.7	1,136	59,777	51.7	6.2	8,037	97.60	95.70
*C. pazoutovae*	CCC1485	Claviceps	Stipeae	*Stipa dregeana*	61×	27.6	1,304	42,785	51.7	6.8	7,941	97.50	96.00
*C. monticola*	CCC1483	Claviceps	Brachypodieae	*Brachypodium sp.*	58×	27.8	1,144	56,619	51.6	7.0	7,977	98.10	96.50
*C. pusilla*	CCC602	Pusillae	Andropogoneae	*Bothriochloa insculpta*	52×	45.9	5,068	15,010	40.4	42.1	8,735	90.90	88.30
*C. lovelessii*	CCC647	Pusillae	Eragostidinae	*Eragrostis* sp.	53×	41.1	5,300	12,480	42.1	33.9	8,862	91.60	88.20
*C. digitariae*	CCC659	Pusillae	Paniceae	*Digitaria eriantha*	57×	33.4	1,773	32,638	44.8	20.0	8,285	95.90	94.70
*C. maximensis*	CCC398	Pusillae	Paniceae	*Megathyrsus maximus*	58×	33.0	829	81,956	44.9	19.8	7,943	98.30	96.50
*C. sorghi*	CCC632	Pusillae	Andropogoneae	*Sorghum bicolor*	60×	35.6	3,660	16,225	44.4	30.4	8,208	89.90	87.10
*C. africana*	CCC489	Pusillae	Andropogoneae	*Sorghum bicolor*	56×	37.7	1,781	37,639	42.5	34.0	8,119	95.00	91.50
*C. citrine*	CCC265	Citrinae	Cynodonteae	*Distichlis spicata*	64×	43.5	4,772	16,294	41.5	51.7	7,821	92.20	88.20

Note.—TE content represented as percent of the genome masked by TEs.

a Newly identified species (Liu et al. 2020).

b The reference strain *C. purpurea* 20.1 was additionally assembled into 191 scaffolds with a scaffold N50 of 433,221.

Overall, species of section *Claviceps* had better assemblies and annotations than species of other sections regarding contig numbers, N50’s, and BUSCO completeness scores (table 1). Nearly all species of section *Claviceps* showed higher BUSCO scores than the references, whereas species of sections *Pusillae* and *Citrinae* generally showed lower scores, likely due to their higher TE content (average 34.9 ± 11.0%, table 1). Exceptions to the low BUSCO scores were *C. digitariae* and *C. maximensis* (sect. *Pusillae*), which had lower TE content, 20.0% and 19.8%, respectively, than the rest of the species in section *Pusillae* (table 1). Although, *C. africana* (sect. *Pusillae*, TE content = 34.0%) also had comparable BUSCO scores, to the references, with a higher N50 and lower contig number, than the rest of the species in section *Pusillae* (table 1). Despite the differences in assembly quality between species of section *Pusillae*, the genomic findings reported in this study were found to be comparable between members of this section indicating that both higher quality and lower quality genomes of section *Pusillae* provided similar results.

### Phylogenomics and Genome Fluidity

**Fig. 1:**
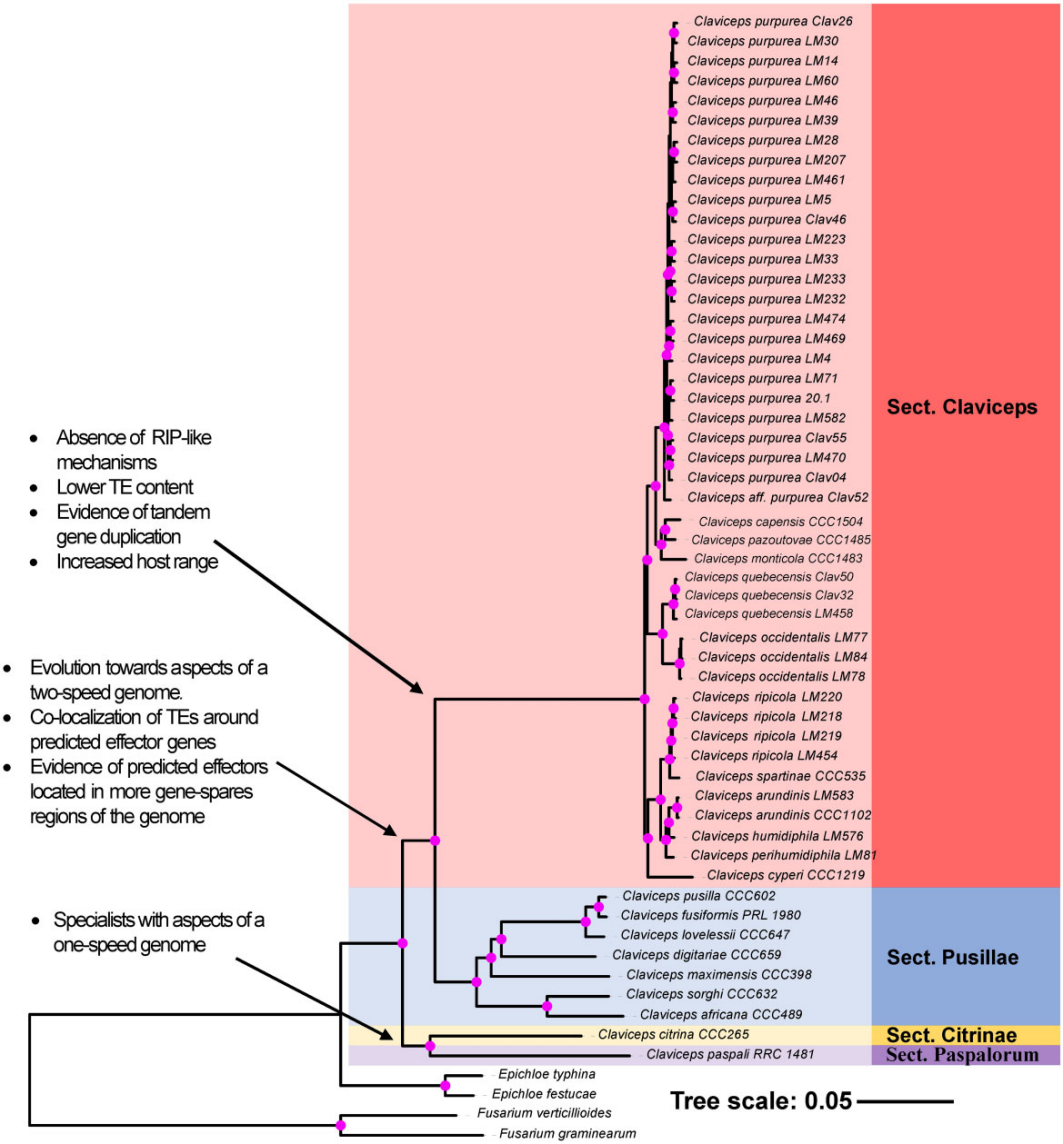
ML phylogenetic reconstruction of the *Claviceps* genus using amino acid sequences of 2,002 single copy orthologs with 1000 bootstrap replicates. Pink dots at branches represent bootstrap values ≥95. Arrows and descriptions indicate potential changes in genomic architecture between *Claviceps* sections identified in this study.

Orthologous gene clusters (orthogroups), which contain orthologs and paralogs, were inferred from protein homology and MCL clustering using OrthoFinder. Across the 53 *Claviceps* isolates and outgroups species *Fusarium graminearum*, *F. verticillioides*, *Epichloe festucae*, and *E. typhina*, we identified 2,002 single-copy orthologs. We utilized a supermatrix approach to infer an ML species tree, based on these protein sequences. Results showed statistical support for four sections of *Claviceps* with a near concordant topology to the Bayesian five-gene phylogeny in Píchová et al. (2018). In addition, our topology of section *Claviceps* is concordant with a larger multilocus phylogeny of the section (Liu et al. 2020). Our ML topology was also supported by NJ and maximum parsimony supermatrix analyses (supplementary fig. S2 and S3, Supplementary Material online). Notable exceptions were the placement of *C. paspali* (sect. *Paspalorum*) which grouped closer to *C. citrina* (sect. *Citrinae*) instead of section *Claviceps*, and *C. pusilla* which grouped closer to *C. fusiformis* instead of *C. maximensis* (fig. 1). We also found that section *Claviceps* diverged from a common ancestor with section *Pusillae* as opposed to section *Paspalorum*. Our results provide support for the deeply divergent lineages of sections *Pusillae*, *Paspalorum*, and *Citrinae* with a long divergent branch resulting in section *Claviceps* (fig. 1).

Each of the 2,002 single-copy orthologs were also independently aligned and analyzed in the same manner as our supermatrix phylogeny from representative isolates of each species. A density consensus tree of all 2,002 topologies was concordant with our supermatrix analysis but reveals evidence of incongruencies, particularly within section *Claviceps* (supplementary fig. S4, Supplementary Material online), which could be caused by biological, analytical, and sampling factors (Steenwyk et al. 2019). Although grouping of species generally held true to figure 1, variation was more related to the order of branches, with *C. cyperi*, *C. arundinis*, *C. humidiphila*, and *C. perihumidiphila* showing the most variability. These results indicate the presence of some incongruencies within section *Claviceps*, section *Pusillae*, and across the genus (supplementary fig. S5–S7, Supplementary Material online) but a consensus supporting our ML species tree (fig. 1 and supplementary fig. S4, Supplementary Material online). There are several potential causes of these incongruencies that are currently the focal point of an ongoing study.

To further elucidate trends of divergence within the genus, we examined genomic fluidity (Kislyuk et al. 2011) using all 82,267 orthogroups from our previous OrthoFinder analysis. Genomic fluidity estimates the dissimilarity between genomes by using ratios of the number of unique orthogroups to the total number of orthogroups in pairs of genomes averaged over randomly chosen genome pairs from within a group on *N* genomes. For example, a fluidity value of 0.05 indicates that randomly chosen pairs of genomes in a group will on average have 5% unique orthogroups and share 95% of their orthogroups (Kislyuk et al. 2011). Section *Claviceps*, which is composed of 12 different species, showed a relatively small genomic fluidity (0.0619 ± 0.0019) with limited variation, indicating pairwise orthogroup dissimilarity between randomly sampled genomes was quite low. The amount of variation between 12 different *Claviceps* species was similar to the variation between 24 *C. purpurea* s.s. isolates, however, the fluidities were significantly different (*P *<* *0.0001; supplementary table S5, Supplementary Material online). In comparison, the fluidity of section *Pusillae* (0.126 ± 0.014; *P *<* *0.0001; supplementary table S5, Supplementary Material online) was two times greater than the fluidity of section *Claviceps* and exhibited greater variation, indicating greater dissimilarities in orthogroups between randomly sampled species of section *Pusillae*.

**Fig. 2:**
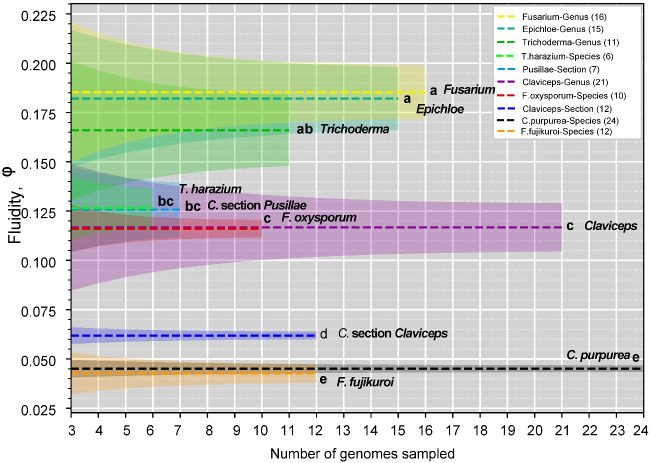
Genomic fluidity (dashed lines) for specified groups within the order Hypocreales. Species level groups contain multiple isolates of a given species, whereas section and genus level groups contain one strain from representative species to remove phylogenetic bias. Shaded regions represent standard error and were determined from total variance, containing both the variance due to the limited number of samples genomes and the variance due to subsampling within the sample of genomes. Letters correspond to significant difference between fluidities determined through a two-sided two-sample *z* test (*P *<* *0.05; supplementary table S4, Supplementary Material online). Legend is in descending order based on fluidity, and names are additionally appended to mean lines for clarity.

Overall, our ML phylogeny (fig. 1) and genome fluidity analysis (fig. 2) indicate a large evolutionary divergence separating section *Claviceps*. Our subsequent analyses of the genomic architecture of all *Claviceps* species examine factors that could be associated with the evolutionary divergence of section *Claviceps* and those driving cryptic speciation.

### TE Divergences and Locations

Due to variation in sequencing platforms that generated the genome data, we examined the relationship of sequence quality with predicted TE content to test for potential biases. Results identified two clusters of genomes with differing sequence qualities, which was determined to be a result of the sequencer used. Although these differences existed, analysis of each cluster showed a lack of relationship between sequence quality and TE content (supplementary fig. S8, Supplementary Material online). In addition, section *Claviceps* samples were sequenced with both sequencers and results were highly comparable between these samples (reported below), indicating no sequence quality bias.

**Fig. 3:**
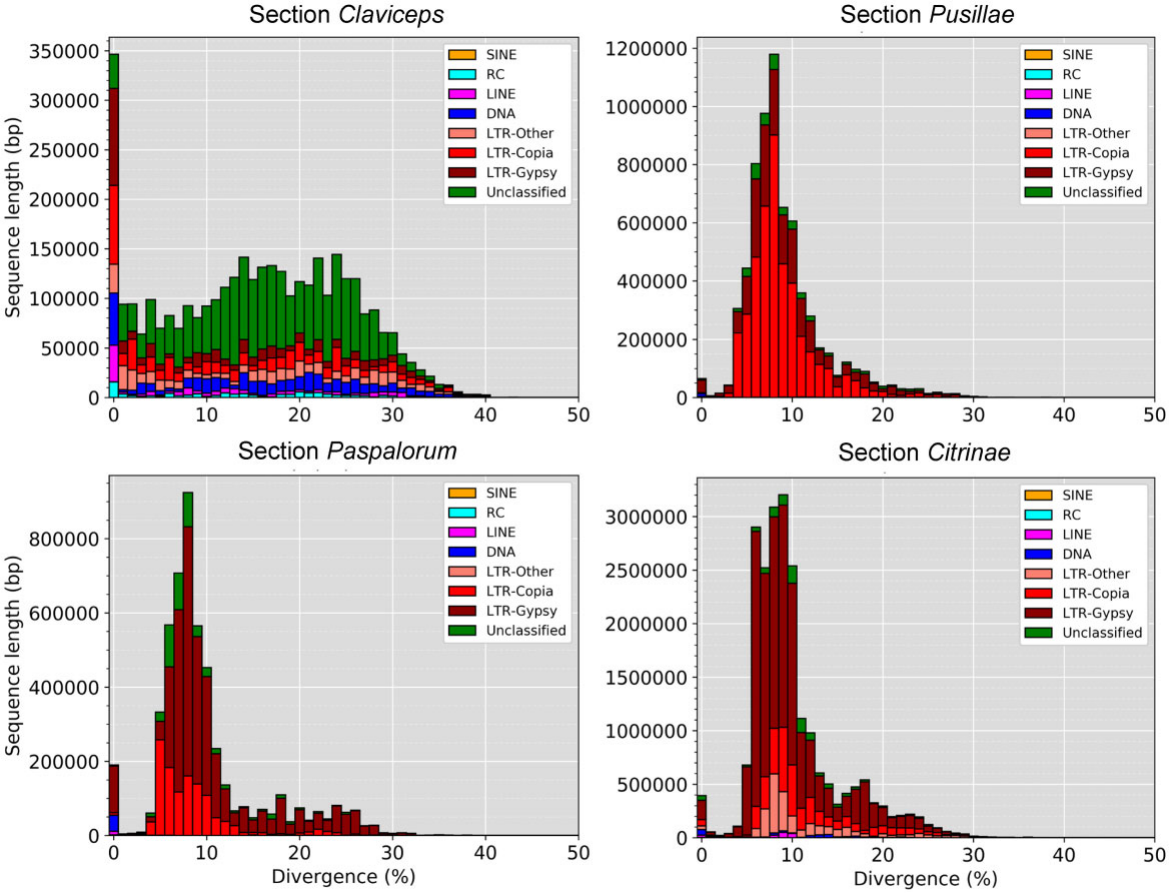
TE fragment divergence landscapes for representative species of each *Claviceps* section; *C. purpurea* 20.1 (sect. *Claviceps*), *C. maximensis* CCC398 (sect. *Pusillae*), *C. paspali* RRC1481 (sect. *Paspalorum*), and *C. citrina* (sect. *Citrinae*). Stacked bar graphs show the nonnormalized sequence length occupied in each genome (*y* axis) for each TE type based on their percent divergence (*x* axis) from their corresponding consensus sequence. Landscape for all remaining isolates can be seen in supplementary figure S8, Supplementary Material online.

TE divergence landscapes revealed an overrepresentation of LTR elements in sections *Pusillae*, *Citrinae*, and *Paspalorum*. All three sections showed a similar large peak of LTRs with divergences between 5% and 10% (fig. 3 and supplementary fig. S9, Supplementary Material online), indicating a relatively recent expansion of TEs. The landscapes of sections *Pusillae*, *Citrinae*, and *Paspalorum* are in striking contrast to species of section *Claviceps* that showed more similar abundances of LTR, DNA, LINE, SINE, and RC (helitron) elements. Species of section *Claviceps* showed broader peaks of divergence between 5% and 30% but also showed an abundance of TEs with ∼0% divergence suggesting very recent TE expansion (fig. 3 and supplementary fig. S9, Supplementary Material online). The TE landscape of *C. cyperi* showed a more striking peak of divergence between 5% and 10% that more closely resembled the TE divergences of sections *Pusillae*, *Paspalorum*, and *Citrinae*. However, the content of the TE peak in *C. cyperi* largely contained DNA, LINE, and unclassified TEs as opposed to LTR’s (supplementary fig. S9, Supplementary Material online).

**Fig. 4:**
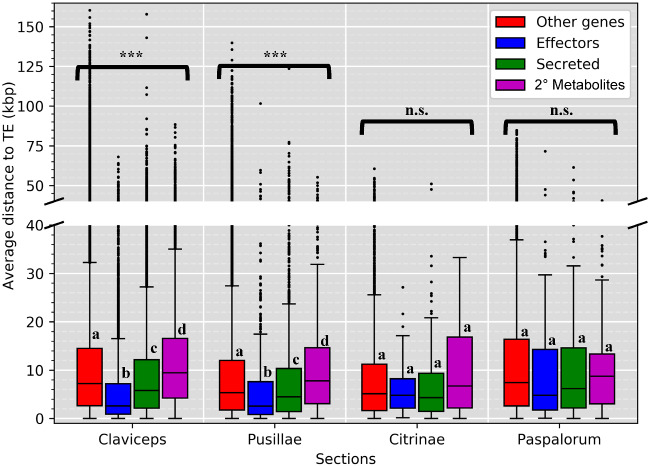
Boxplot distributions of predicted effectors, secreted (noneffectors), secondary metabolite (nonsecreted) genes, and other genes (i.e., genes that are not effectors, secreted, or secondary [2°] metabolite genes) in *Claviceps* sections showing the mean distance (kb) of each gene to the closest TE fragment (5′ and 3′ flanking distances were averaged together). Kruskal–Wallis (*P* value: *<0.05, **<0.01, ***<0.001, n.s. = not significant). Pairwise comparison was performed with Mann–Whitney *U* test with Benjamini–Hochberg multitest correction. Letters correspond to significant differences between gene categories within sections (*P *<* *0.05). Plots for all individual isolates can been seen in supplementary figure S9, Supplementary Material online.

To identify where genes were located in relation to TEs, we calculated the average distance (kb) of each gene to the closest TE fragment. This analysis was performed for predicted effectors, secreted (noneffector) genes, secondary metabolite (nonsecreted) genes, and all other genes. Secreted genes and predicted effectors of sections *Claviceps* and *Pusillae* species were found to be significantly closer to TEs compared with other genes within each respective section (fig. 4; *P *<* *0.0001), suggesting that these genes could be located in more repeat-rich regions of the genome. It should be noted that we did observe a significant difference (*P *<* *0.001, Welch’s test) in TE content between section *Pusillae* (32.5 ± 9.59%) and section *Claviceps* (8.79 ± 1.52%). In both sections *Claviceps* and *Pusillae*, secondary metabolite genes were located farther away from TEs (fig. 4; *P *<* *0.0001), that is, repeat-poor regions of the genome. These trends hold true for individual isolates, with a notable exception of *C. pusilla* (sect. *Pusillae*) showing no significant differences in the proximity of TEs to specific gene types (*P *>* *0.12; supplementary fig. S10, Supplementary Material online). Variation existed in whether particular isolates had significant differences between all other genes compared with secreted genes and secondary metabolite genes, but all species in sections *Claviceps* and *Pusillae* (aside from *C. pusilla*) had predicted effector genes located significantly closer to TEs (*P *<* *0.003; supplementary fig. S10, Supplementary Material online). No significant differences in the proximity of TEs to specific gene types were observed in sections *Citrinae* and *Paspalorum* (fig. 4; *P *>* *0.11), suggesting that TE’s are more randomly distributed throughout these genomes.

### Gene Density Compartmentalization

**Fig. 5:**
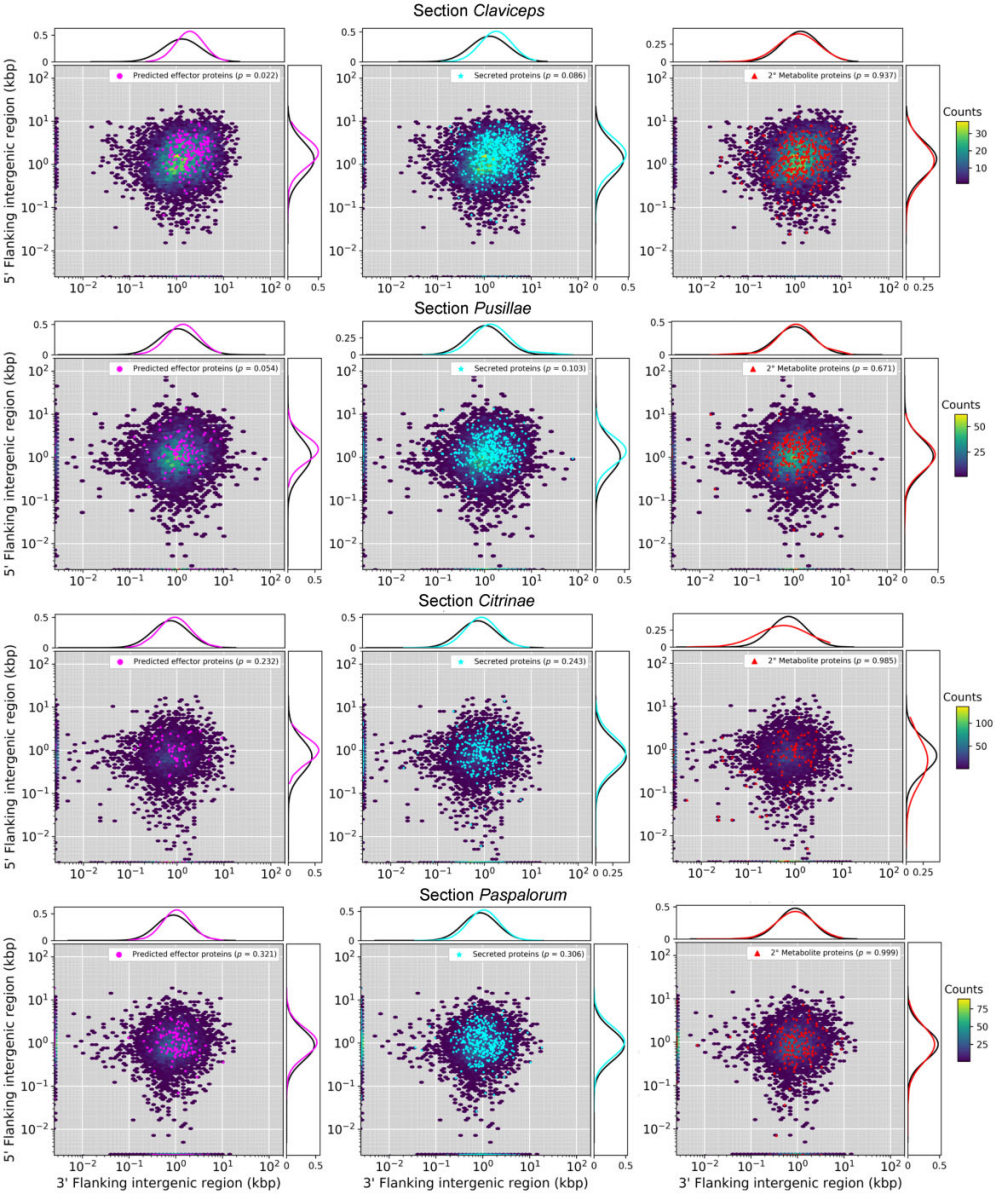
Gene density as a function of flanking 5' and 3' intergenic region size (*y*- and *x* axis) of representative isolates of each of the four sections within the *Claviceps* genus; *C. purpurea* 20.1 (sect. *Claviceps*), *C. maximensis* CCC398 (sect. *Pusillae*), *C. paspali* RRC1481 (sect. *Paspalorum*), and *C. citrina* (sect. *Citrinae*). Colored hexbins indicate the intergenic lengths of all genes with color code indicating the frequency distribution (gene count) according to the legend on the right. Overlaid markers indicate specific gene types corresponding to legends in the top right within each plot. Line graphs (top and right of each plot) depict the frequency distributions of specific gene types (corresponding legend color) and all other genes not of the specific type (black). For visualization purposes, the first genes of contigs (5′ end) are plotted along the *x* axis and the last gene of each contig (3′ end) are plotted along the *y* axis. For information on statistical test, see Methods and for plots of all remaining isolates see supplementary figure S10, Supplementary Material online.

To further examine genome architecture, we analyzed local gene density measured as flanking distances between neighboring genes (intergenic regions) to examine evidence of gene density compartmentalization (i.e., clustering of genes with differences in intergenic lengths) within each genome. Results showed that all 53 *Claviceps* strains exhibited a one-compartment genome (lack of multiple compartments of genes with different intergenic lengths). Although, there was a tendency for more genes with larger intergenic regions in sections *Claviceps* and *Pusillae* compared with sections *Citrinae* and *Paspalorum* (fig. 5; supplementary fig. S11, Supplementary Material online).

To further clarify evolutionary tendencies, we evaluated whether gene types showed a difference in their flanking intergenic lengths compared with other genes within their genomes. Results showed that predicted effector genes in section *Claviceps* had significantly larger intergenic flanking regions compared with other genes, indicating they may reside in more gene-sparse regions of the genome (*P *<* *0.04, fig. 5, supplementary fig. S11, Supplementary Material online). Only *C. digitariae* and *C. lovelessi* (*P *<* *0.01, *P *=* *0.024, respectively; supplementary fig. S11, Supplementary Material online) of section *Pusillae* had predicted effector genes with significantly larger intergenic regions than other genes, although *C. fusiformis* and *C. pusilla* were near significant (fig. 5, *P *=* *0.054, *P *=* *0.056, respectively; supplementary fig. S11, Supplementary Material online). Flanking intergenic lengths of secreted genes also showed larger intergenic lengths and were often significantly larger than other genes in section *Claviceps* (fig. 5; supplementary fig. S11, Supplementary Material online). In contrast, secondary metabolite genes exhibited a widespread distribution of intergenic lengths that were not significantly different than other genes in all 53 *Claviceps* strains (*P *>* *0.55, fig. 5; supplementary fig. S11, Supplementary Material online).

### RIP Analysis

**Fig. 6:**
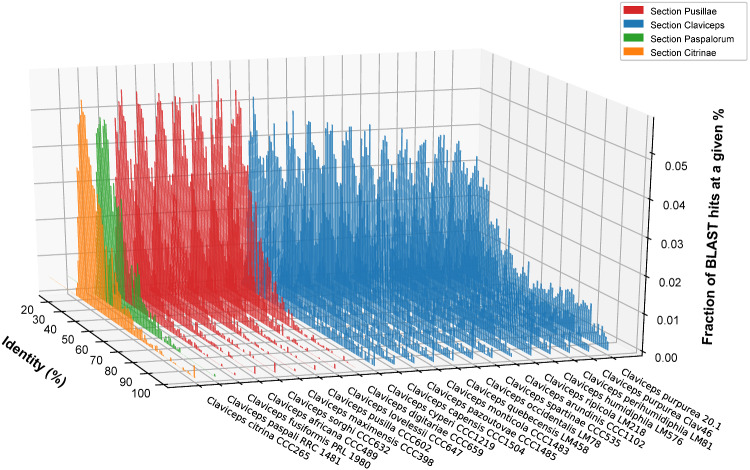
Representative isolates of each *Claviceps* species showing the fraction of Blast hits at a given % identity (*y* axis) within each isolate (*z* axis) at a given percent identity (*x* axis) from the second closet BlastP match of proteins within each isolate’s own genome. Two *C. purpruea* s.s. isolates are shown to compare a newly sequenced genome versus the reference.

To test for effects of RIP-like signatures, we assessed the bidirectional similarity of genes against the second closest BlastP match within each isolate’s own genome (Galagan et al. 2003; Urguhart et al. 2018), supported by a BlastP analysis against the *rid-1* RIP gene of *Neurospora crassa*, and calculations of RIP indexes in 1-kb windows (500 bp increments) using The RIPper (Van Wyk et al. 2019). Results showed that sections *Pusillae*, *Citrinae*, and *Paspalorum* had homologs of *rid-1*, fewer genes with close identity (≥80%), on average 27.4 ± 11.4% of their genomes affected by RIP, a mean RIP composite index of −0.03 ± 0.21, and 325 ± 138 LRARs covering 3,984 ± 2,144 kb of their genomes, indicating past or current activity of RIP-like mechanisms (fig. 6; supplementary tables S6–S8, Supplementary Material online). This is further supported by an average GC content of 42.84 ± 3.03% (table 1) in sections *Pusillae*, *Citrinae*, and *Paspalorum*, which is on average 8.81% lower than in section *Claviceps* that shows an absence of RIP (reported below). The presence of RIP-like mechanisms in sections *Pusillae*, *Citrinae*, and *Paspalorum* was unexpected, given the abundance of TEs within genomes of these sections (table 1, fig. 3, and supplementary fig. S9, Supplementary Material online) as RIP-like mechanisms should be working to silence and inactivate these TEs. Although we did not directly test the activity of TEs within our genomes, due to lack of RNAseq data, the peaks of low TE nucleotide divergence (<10%) in sections *Pusillae*, *Citrinae*, and *Paspalorum* (fig. 3, supplementary fig. S9, Supplementary Material online) suggest recent activity of TEs (Frantzeskakis et al. 2018).

In comparison, species in section *Claviceps* lack *rid-1* homologs, showed larger amounts of gene similarity, and a general lack of evidence of RIP-like signatures with only 0.13 ± 0.03% of their genomes putatively affected by RIP, and a mean RIP composite index of −0.59 ± 0.01 suggesting that RIP-like mechanisms are inactive (fig. 6 and supplementary tables S6–S8, Supplementary Material online). Gene pairs sharing a ≥80% identity to each other were often located near each other. On average 27.02 ± 5.91% of the pairs were separated by five or fewer genes, and 15.95 ± 3.50% of the pairs were located next to each other, indicating signs of tandem gene duplication within the section (supplementary table S6, Supplementary Material online). *C. cyperi* showed the smallest proportions of highly similar tandem genes (7.77% and 5.7%) compared with other species within section *Claviceps*. Additional variations in the proportions of highly similar tandem genes between other species of section *Claviceps* were not evident as these proportions appeared to vary more between isolate than species (supplementary table S6, Supplementary Material online).

### Gene Cluster Expansion

The proteome of *Claviceps* genomes were used to infer orthologous gene clusters (orthogroups) through protein homology and MCL clustering using OrthoFinder. Our results revealed evidence of orthogroup expansion within section *Claviceps* as species contained more genes per orthogroup than species of the other three sections (supplementary fig. S12, Supplementary Material online). To identify the types of gene clusters that were showing putative expansion, we filtered our clusters by following two criteria: 1) at least one isolates had two or more genes in the orthogroup and 2) there was a significant difference in the mean number of genes per orthogroup between all 44 isolates in section *Claviceps* and the 9 isolates from sections *Pusillae*, *Citrinae*, and *Paspalorum* (*α* ≤ 0.01, Welch’s test).

**Fig. 7:**
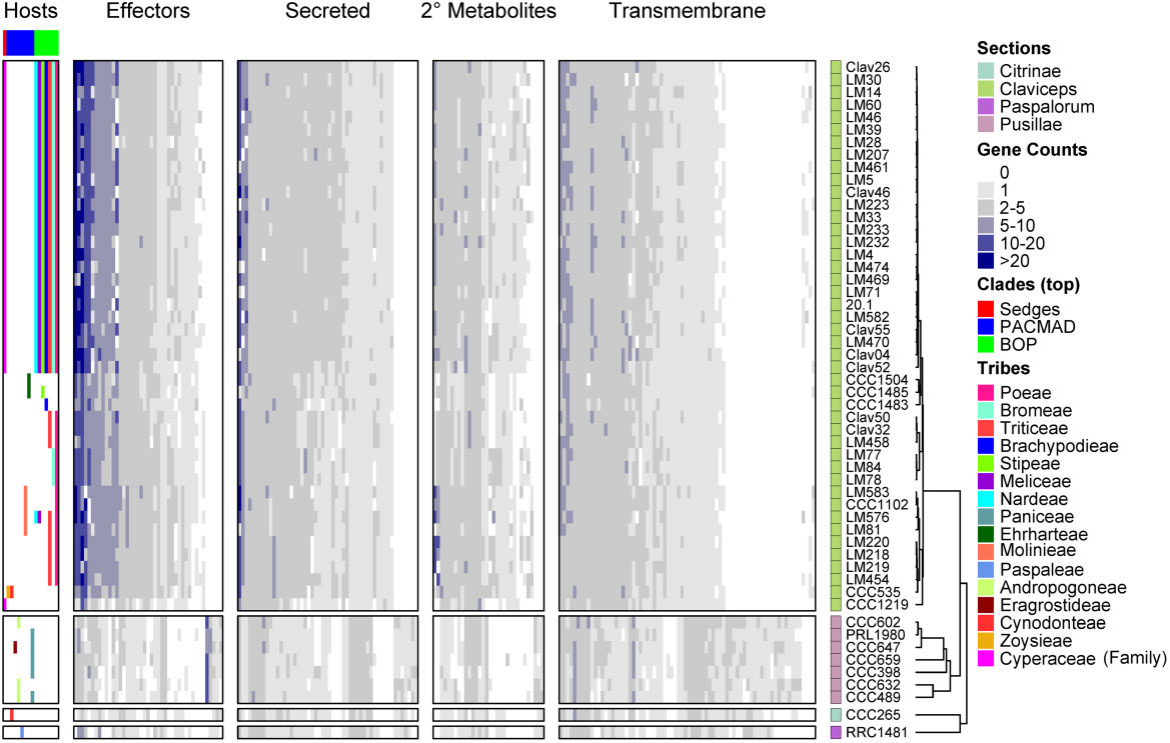
Heatmap of gene counts in orthogroups for all 53 *Claviceps* strains ordered based on ML tree in figure 1 and separated by sections. Orthogroups are separated based on their classification and are only represented once (i.e., secondary [2°] metabolite orthogroups shown are those that are not already classified into the effector or secreted orthogroups) and are ordered based on hierarchical clustering, see supplementary table S9, Supplementary Material online, for list of orthogroups corresponding to the order shown in the heatmaps. The host spectrum (right) is generalized across species, as no literature has determined the existence of race specific isolates within species, is shown on the left side of the figure determined from literature review of field collected samples (Supplementary Material in Píchová et al. 2018) and previous inoculation tests Campbell (1957) and Liu et al. (2020). For heatmap of conserved domains, see supplementary figure S12, Supplementary Material online, and for unclassified gene families, see supplementary figure S13, Supplementary Material online.

Overall, we identified 863 (4.7%) orthogroups showing putative expansion. We observed extensive expansion (orthogroups with observations of greater than or equal to ten genes per isolate) present in many unclassified, predicted effectors, secreted (noneffector) orthogroups, and orthogroups encoding genes with conserved domains (fig. 7 and supplementary figs. S13 and S14, Supplementary Material online). Transmembrane orthogroups also showed evidence of expansion with several isolates having five to ten genes. Orthogroups with secondary metabolite genes showed the lowest amount of expansion (supplementary fig. S15, Supplementary Material online). Overall, section *Claviceps* showed expansion in a greater number of orthogroups than section *Pusillae*, *Citrinae*, and *Paspalorum* in all categories except transmembranes (supplementary fig. S15, Supplementary Material online). Orthogroups with an average greater than or equal to five genes per isolate, within section *Claviceps*, contained a variety of functional proteins, with generally more proteins encoding protein/serine/tyrosine kinase domains (supplementary table S9, Supplementary Material online). Additional details can be obtained from supplementary tables S10 (ordered orthogroups corresponding to heatmaps; fig. 7 and supplementary figs. S13 and S14, Supplementary Material online), S11-1, and S11-2, Supplementary Material online (orthogroups identification and functional annotation of all proteins).

Within section *Claviceps* patterns of gene counts per orthogroup appeared to break down and contain variations in the number of genes per orthogroups with some presence/absences occurring between isolates and species. Notably, *C. cyperi* (CCC1219) showed the lowest amount of expansion, across all taxa, in comparison with other species of section *Claviceps*. In addition, *C. spartinae* (CCC535)*, C. capensis* (CCC1504), *C. monticola* (CCC1483), *C. pazoutovae* (CCC1485), *C. occidentalis* (LM77, 78, 84), and *C. quebecensis* (LM458, Clav32, 50) also showed lower expansion (fig. 7, supplementary figs. S13 and S14, Supplementary Material online). However, no patterns were observed linking the variation in expansions with the literature determined host range of different species within section *Claviceps*.

## Discussion

Our comparative study of 50 newly annotated genomes from four sections of *Claviceps* has provided us with an enhanced understanding of evolution in the genus through knowledge of factors associated with its diversification. Our results have revealed that despite having nearly identical life strategies, these closely related species have substantially altered genomic architecture and plasticity, which may drive genome adaptation. One key difference we observe is a shift from aspects that are characteristic of a one-speed genome (i.e., less adaptable) in narrow host-range *Claviceps* species (sects. *Citrinae* and *Paspalorum*) toward aspects that are characteristic of a two-speed genome (i.e., more adaptable) in broader host-range lineages of sections *Pusillae* and *Claviceps* (fig. 1; Dong et al. 2015; Frantzeskakis et al. 2019).

The oldest divergent species of the genus (Píchová et al. 2018), *C. citrina* (sect. *Citrinae*) and *C. paspali* (sect. *Paspalorum*), are characterized by a proliferation of TEs, particularly LTRs, which do not appear to be colocalized around particular gene types (fig. 4). Coupled with a lack of large-scale genome compartmentalization (fig. 5), these two species can be considered to fit with aspects of a one-speed genome which are often considered to be less adaptable and potentially more prone to being purged from the biota (Dong et al. 2015; Frantzeskakis et al. 2019). This could help explain the paucity of section lineages and restricted host range to one grass tribe, as similar patterns of large genome size, abundant TE content, and equal distribution of TEs has been observed in the specialized barley pathogen *Blumeria graminis* f.sp. *hordei* (Frantzeskakis et al. 2018). Although, rapid adaptive evolution within *B. graminis* f.sp. *hordei*, has been suggested to occur through copy-number variation and/or heterozygosity of effector loci (Dong et al. 2015; Frantzeskakis et al. 2018, 2019). Our results show a lack of gene duplication occurring in sections *Citrinae* and *Paspalorum* likely due to the presence of RIP-like mechanisms. However, even with the presence of RIP-like mechanisms, there was a high LTR content in these species (fig. 3). This suggests that these LTR elements have found a way to avoid RIP-like mechanisms or indicate that these species harbor a less active version of an RIP-like mechanisms as is found in several fungal species (Kachroo *et al.* 1994; Nakayashiki et al. 1999; Graïa et al. 2001; Ikeda et al. 2002; Chalvet et al. 2003; Kito et al. 2003). Nonetheless, due to the high abundance of TEs (fig. 4) and presence of RIP (fig. 6 and supplementary tables S6 and S7, Supplementary Material online), we hypothesize that aspects of RIP-like “leakage” could be a likely mechanism for evolution in *C. citrina* and *C. paspali* (and similarly sect. *Pusillae*) as has been shown to occur in other fungi (Fudal et al. 2009; Van de Wouw et al. 2010; Hane et al. 2015). It should be noted that since the estimated divergence of section *Citrinae* 60.5 Ma (Píchová et al. 2018), it has remained monotypic. It was only recently that unknown lineages of section *Paspalorum* were identified (Oberti et al. 2020), although these lineages were found on the same genera of host as *C. paspali* (*Paspalum* spp.) supporting our hypothesis that species within section *Paspalorum* have restricted host ranges. These recent findings further suggest that lack of additional lineages within these sections could be due to limited records of *Claviceps* species in South America, where the genus is thought to have originated (Píchová et al. 2018). Further research into South American populations of *Claviceps* will provide significant insight into the evolution of these two sections.

Members of section *Pusillae* also exhibited a proliferation of TEs, however, as this section diverged from sections *Citrinae* and *Paspalorum*, the genomic architecture evolved such that TEs colocalized around predicted effector genes (fig. 4). This proximity of TEs to effectors persisted in section *Pusillae* species (except *C. pusilla*; supplementary fig. S10, Supplementary Material online) and section *Claviceps* species potentially resulting in the large intergenic regions flanking predicted effector genes (fig. 5, supplementary fig. S11, Supplementary Material online). Together, these genomic alterations indicate aspects of a two-speed genome (Dong et al. 2015; Möller and Stukenbrock 2017). These observed genomic changes may have influenced the divergence and adaptability of sections *Pusillae* and *Claviceps* (fig. 1) similar to what has been observed in other fungi (Raffaele and Kamoun 2012; Stukenbrock 2013; Möller and Stukenbrock 2017) and has been proposed to promote genomic flexibility and drive accelerated evolution of these genome compartments (Raffaele et al. 2010; Rouxel et al. 2011; de Jonge et al. 2013; Faino et al 2015, 2016; Seidl et al. 2015). Despite the number of studies that suggest this role of TEs in genome evolution, there has been limited evidence for the mechanism by which TEs drive evolution in filamentous pathogens. However, studies incorporating improved genome assemblies of multiple individuals of a species along with transcriptome data have been able to demonstrate that transcriptionally active TEs were observed in lineage-specific regions of the plant pathogen *Verticillium dahliae* (Amyotte et al. 2012; Faino et al. 2016), resulting in genomic diversity through large scale duplications in these lineage-specific regions (Faino et al. 2016). This also lead to the frequent loss of the effector *Ave1* in populations of *V. dahliae*, which is located in a TE-rich lineage-specific region (de Jonge et al. 2012).

Although we did not have transcriptome data to determine how many of the TEs are transcriptionally active, our data do show that most of the repetitive elements in section *Claviceps* species have very low nucleotide divergence (<1%) compared with TEs in sections *Pusillae*, *Paspalorum*, and *Citrinae* (5–20% nucleotide divergence; fig 3), suggesting a recent section specific expansion of TEs that are associated with a recent host range and geographic expansion and proliferation of recently described cryptic species (Liu et al. 2020) within section *Claviceps*. Similar observations placing TE bursts around speciation times have been reported in the plant pathogen *Leptosphaeria maculans* (Rouxel et al. 2011; Grandaubert et al. 2014), and the grass-infecting (*Blumeria* spp.) and dicot-infecting (*Erysiphe* spp.) powdery mildews (Frantzeskakis et al. 2018). Theoretical models have proposed that repeated changes in phenotypic optimum in a dynamic fitness landscape may induce explosive bursts of transposon activity associated with faster adaptation (Startek et al. 2013). However, long-term maintenance of transposon activity is unlikely, and this may contribute to significant variation in the TE copy number among closely related species. Our findings that the variation in TE copy number between species in the genus *Claviceps* fits this pattern and call for future studies to clarify the relationship between TE expansion and changes in host range, geographic distribution, and cryptic speciation.

Furthermore, our analyses revealed that a key difference between section *Claviceps* and section *Pusillae* is a putative loss of RIP-like mechanisms (figs. 1, 6 and supplementary table S7, Supplementary Material online). In the absence of RIP-like mechanisms, the gene-sparse regions rich in TEs, and effectors could be hot spots for duplication, deletion, and recombination (Galagan et al. 2003; Galagan and Selker 2004; Raffaele and Kamoun 2012; Dong et al. 2015; Faino et al. 2016; Möller and Stukenbrock 2017; Frantzeskakis et al. 2018, 2019). This would explain the observations of tandem gene duplication within the section (figs. 6, 7 and supplementary table S6, figs. S12–S15, Supplementary Material online), which may facilitate rapid speciation, as has been postulated in several smut fungi (Kämper et al. 2006; Schirawski et al. 2010; Dutheil et al. 2016). In fact, *C. cyperi*, a species of section *Claviceps* and thought to be ancestral from ancestral state reconstructions of host range (Píchová et al. 2018), showed the least amount of gene cluster expansion and tandem duplication (fig. 7 and supplementary table S6, figs. S13 and S14, Supplementary Material online), indicating that gene duplication may be contributing to the divergence of new species, as other species in section *Claviceps* have increased genome size, gene count, and number of closely related gene pairs (≥80% identity) (table 1 and supplementary table S6, Supplementary Material online). It is unclear if these changes in gene duplication rate are a selective or neutral mutational process. Because the increased occurrence of gene duplication within section *Claviceps* is likely a result of a loss of RIP-like mechanisms, it is more plausible to suggest that the change in propensity for gene duplication was a neutral process. However, our evidence of effector duplications suggests that this change in propensity may have allowed an increase chance for future adaptive events. Within section *Claviceps* gene duplication is likely facilitated by recombination events during annual sexual reproduction (Esser and Tudzynski 1978). Future studies on recombination will be critical to our understanding of the mechanisms driving gene duplication and elucidating factors associated with the observations of potential incomplete lineage sorting (Pease and Hahn 2013) within the section.

Substantially altered genomic architecture and plasticity between *Claviceps* sections was observed in this study, yet it is unclear whether the evolution of these genomes were caused by contact with new hosts and different climates as ancestral lineages migrated out of South America (Píchová et al. 2018) or if the evolution toward aspects of a two-speed genome provided an advantage in adapting to new hosts or environments. Further research is needed to clarify this point. As sections *Pusillae* and *Claviceps* have larger host ranges (5 tribes and 13 tribes, respectively) and increased levels of speciation (Píchová et al. 2018), they represent ideal systems to test this hypothesis. It is postulated that section *Pusillae* was transferred to Africa (ca. 50.3 Ma), whereas section *Claviceps* originated in North America (ca. 20.7 Ma), and it is likely that the common ancestor shared between these sections (fig. 1) had strains that were transferred to Africa likely due to insect vectors via transatlantic long-distance dispersal (Píchová et al. 2018). The strains that remained, in South America, likely persisted but appeared to not speciate for roughly 30 Ma (Píchová et al. 2018), despite having aspects of a more adaptable two-speed genome (figs. 4, 5). Limited sampling records could be a factor contributing to this lack of speciation during this 30 Myr period, but it could also be suggested that the ancestral species of sections *Claviceps* did not diverge due to a lack of diversification of host species (Píchová et al. 2018). It is well known that *Claviceps* species share a rather unique relationship with their hosts (strict ovarian parasites). The evolution of the *Claviceps* genus appears to be primarily driven by the evolution and diversification of the host species (Píchová et al. 2018). This can be inferred from divergence time estimates which show that the crown node of section *Pusillae* aligns with the crown node of PACMAD grasses (ca. 45 Ma) (Bouchenak-Khelladi et al. 2010; Píchová et al. 2018), suggesting that these two organisms radiated in tandem after ancestral strains of section *Pusillae* were transferred to Africa. Similarly, the estimated crown node of section *Claviceps* corresponds with the origin of the core Pooideae (Poeae, Triticeae, Bromeae, and Littledaleae), which occurred in North America (ca. 33–26 Ma) (Bouchenak-Khelladi et al. 2010; Sandve and Fjellheim 2010).

Such a large difference between the estimate divergence age (∼30 Myr) and long divergence branch (fig. 1) between section *Clavcieps* and the other three sections (Píchová et al. 2018) could suggest that a sudden event sparked the adaptive radiation within this section (fig. 1). Under an assumption that ancestral strains of section *Claviceps* were infecting sedges (Cyperaceae), as is seen in the ancestral *C. cyperi* (Píchová et al. 2018), a host jump to BOP grasses could have ignited the rapid speciation of section *Claviceps*, similar to the suggested tandem radiation of section *Pusillae* with the PACMAD grasses in Africa. However, unknown factors might be responsible for the drastic genomic changes (i.e., putative loss of RIP-like mechanisms) observed in section *Claviceps*, as no such changes were observed in section *Pusillae*. The radiation of the core Pooideae occurred after a global supercooling period (ca. 33–26 Ma) in North America. During this period, Pooideae experienced a stress response gene family expansion that enabled adaptation and diversification to cooler, more open, habitats (Kellogg 2001; Sandve and Fjellheim 2010). As gene cluster expansion was observed in section *Claviceps* (the only section to infect BOP grasses), it suggests that the same environmental factors that caused the radiation of Pooideae could have similarly affected section *Claviceps* (Kondrashov 2012) and might have resulted in the host jump to Pooideae, and potentially other BOP tribes. Interestingly, one of the orthogroups significantly expanded in section *Claviceps* (OG0000016) contains proteins associated with a cold-adapted (Alias et al. 2014) serine peptidase S8 subtilase (MER0047718; S08.139) (supplementary table S9, Supplementary Material online). Although the crown node of section *Claviceps* is estimated at ∼5–10 Myr before the radiation of the core Pooideae, the 95% highest posterior density determined in Píchová et al. (2018) could indicate both radiation events occurred at similar times.

Further examination of *Claviceps* species in South and Central America needs to be conducted to better elucidate the evolution and dispersal of the genus (Píchová et al. 2018). Efforts should focus on the elusive *C. junci*, a pathogen of Juncaceae (rushes), which is thought to reside in section *Claviceps* based on morphological and geographic characteristics (Langdon 1952; Píchová et al. 2018). This species, and potentially others, will provide further insight into the early evolution of section *Claviceps* and could bridge the current gap between the environmental factors that sparked the radiation of the core Pooideae and section *Claviceps*. Last, it would be interesting to examine if other phytopathogenic fungal species that diverged in North America ∼20 Ma experienced similar genomic alterations and host range expansions.

## Supplementary Material

Supplementary data are available at *Genome Biology and Evolution* online.

## Supplementary Material

evaa267_Supplementary_Data
