# Supplementary material for: Whole-Genome Comparisons of Ergot Fungi Reveals the Divergence and Evolution of Species within the Genus Claviceps Are the Result of Varying Mechanisms Driving Genome Evolution and Host Range Expansion
Source: Genome Biol Evol. 2021 Jan 29;13(2):evaa267. doi: 10.1093/gbe/evaa267 (PMC7883665; doi:10.1093/gbe/evaa267)
Supplement: evaa267_Supplementary_Data [file evaa267_supplementary_data.zip › Additional_File_1_Fig_S1-S15_Table_S1-S7.pdf]

Supplemental Information for:

Whole genome comparisons of ergot fungi reveals the divergence and evolution of species within the genus *Claviceps* are the result of varying mechanisms driving genome evolution and host range expansion

Stephen A. Wyka[1], Stephen J. Mondo[1,2], Miao Liu[3], Jeremy Dettman[3], Vamsi Nalam[1], Kirk D. Broders[4*]

<u>Table of Contents:</u>

**Figure S1**	Page 2	Mean functional proteins per sections
**Figure S2**	Page 3	Neighbor-joining super-matrix phylogeny
**Figure S3**	Page 4	Maximum parsimony super-matrix phylogeny
**Figure S4**	Page 5	Density consensus of gene trees
**Figure S5**	Page 6	*Claviceps* genus tree topology frequency
**Figure S6**	Page 7	*Claviceps* section *Claviceps* tree topology frequency
**Figure S7**	Page 8	*Claviceps* section *Pusillae* tree topology frequency
**Figure S8**	Page 9	Regressions of sequence quality versus TE content
**Figure S9**	Page 10	Transposable elements divergence landscapes
**Figure S10**	Page 11	Distance of genes to closest transposable elements
**Figure S11**	Page 12	Hexbin plots of intergenic flanking regions
**Figure S12**	Page 13	Mean number of paralogs per orthogroups across sections
**Figure S13**	Page 14	Heatmap of orthogroups with conserved domains
**Figure S14**	Page 15	Heatmap of unclassified orthogroups
**Figure S15**	Page 16	Number of orthogroups with expansion per section
**Table S1**	Page 17	Collection and accession information of isolates
**Table S2**	Page 18	Functional protein numbers per isolate
**Table S3**	Page 19	Additional genomes used in OrthoFinder analysis
**Table S4**	Page 20	Count of genes removed from gene density analysis
**Table S5**	Page 21	*P*-values from genomic fluidity analysis
**Table S6**	Page 22	Number of duplicated gene and percent in tandem
**Table S7**	Page 23	RIP-index results from The RIPper

Additional files not located in this file.

<u>Additional File 2:</u>

**Table S8**	BLASTp results of 55 important *Claviceps* genes, including *rid-1* homolog
**Table S9**	Protein domain association of highly expanded orthogroups
**Table S10**	Order of orthogroups displayed in heatmaps

<u>Additional File 3:</u>

**Table S11-1**	All orthogroups with corresponding size, strains, gene IDs, and functional proteins
**Table S11-1**	classification, broken up into two files due to size.

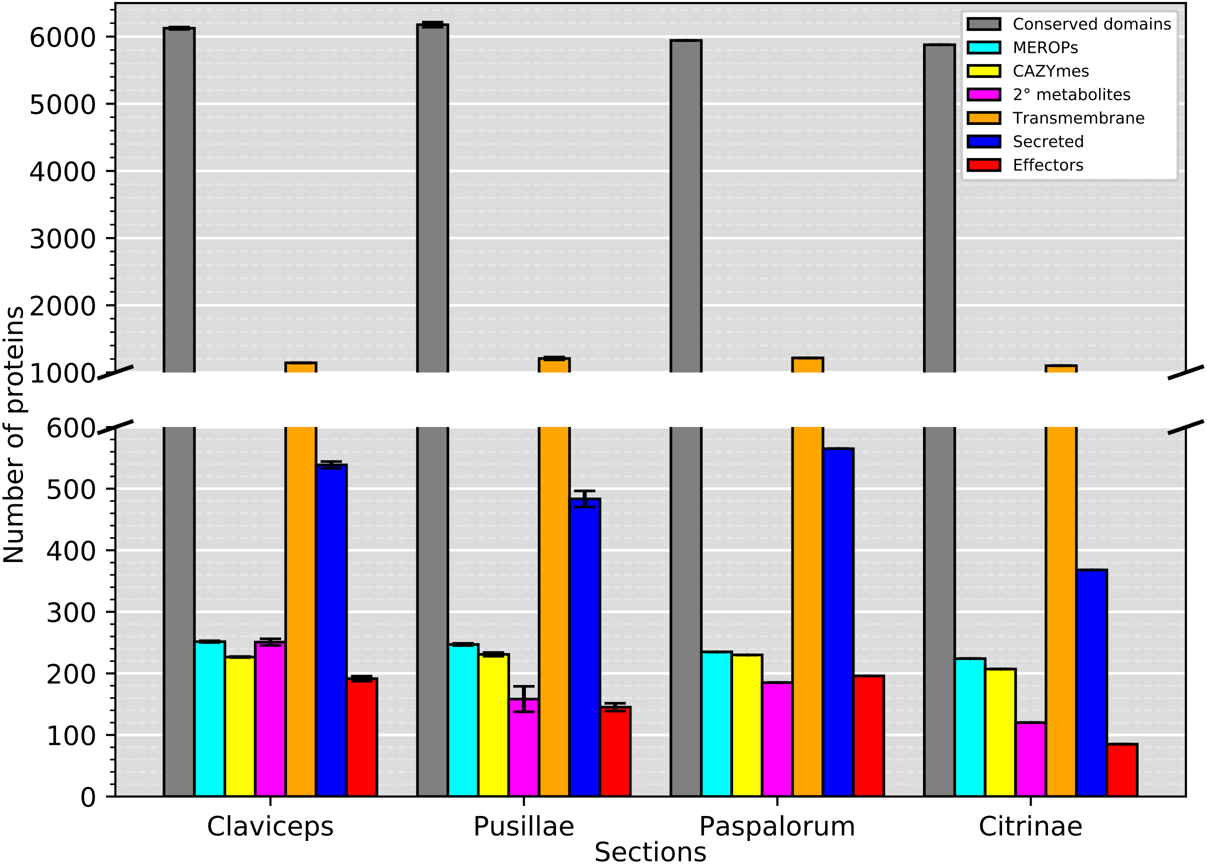

Figure S1: Mean number of proteins in each section of the genus *Claviceps*. Bars represent standard error.

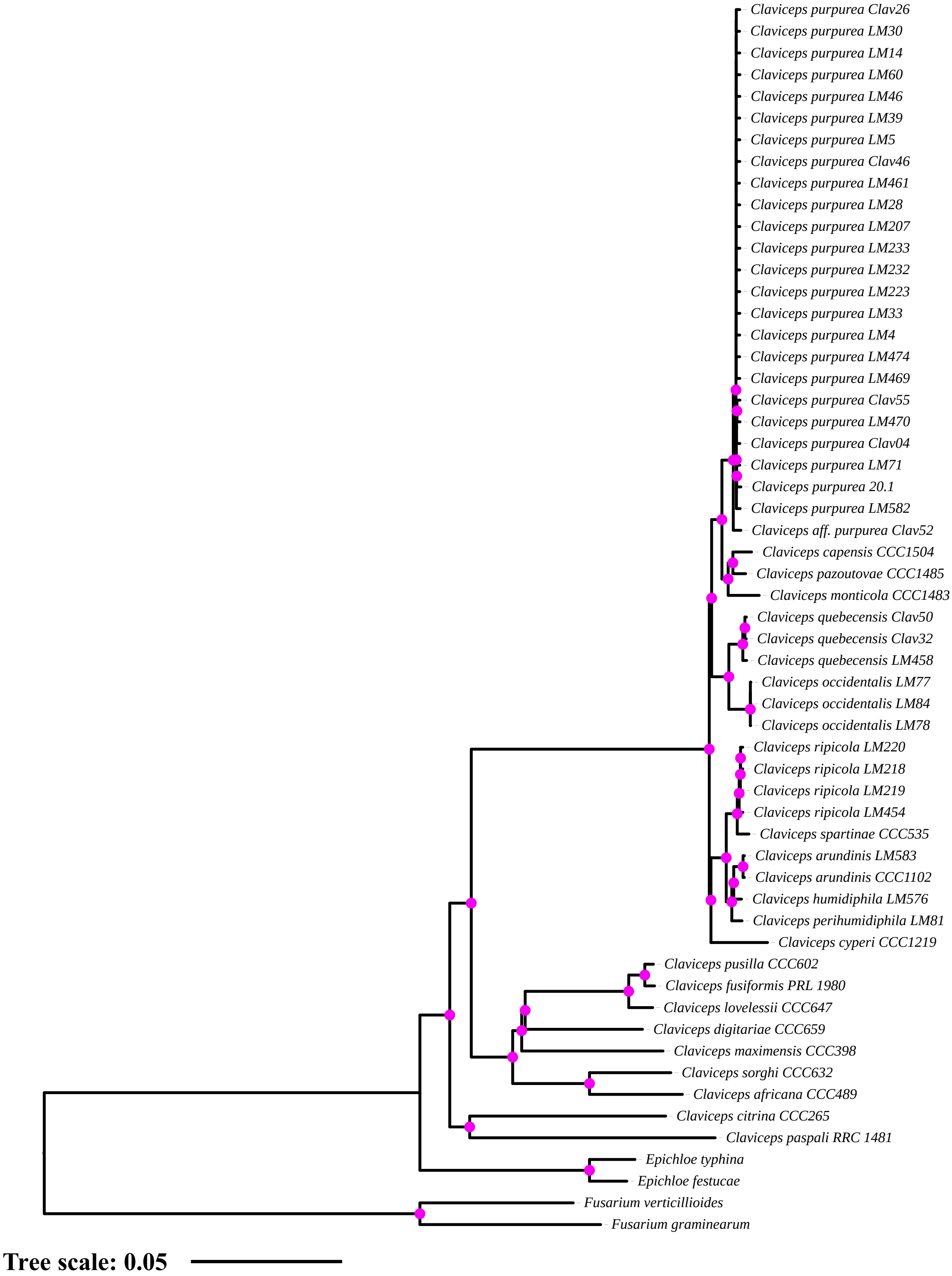

Figure S2: Neighbor-joining phylogenetic reconstruction of the *Claviceps* genus using amino acid sequences of 2,002 single copy orthologs with 1000 bootstrap replicates. Pink dots at branches represent bootstrap values ≥ 95.

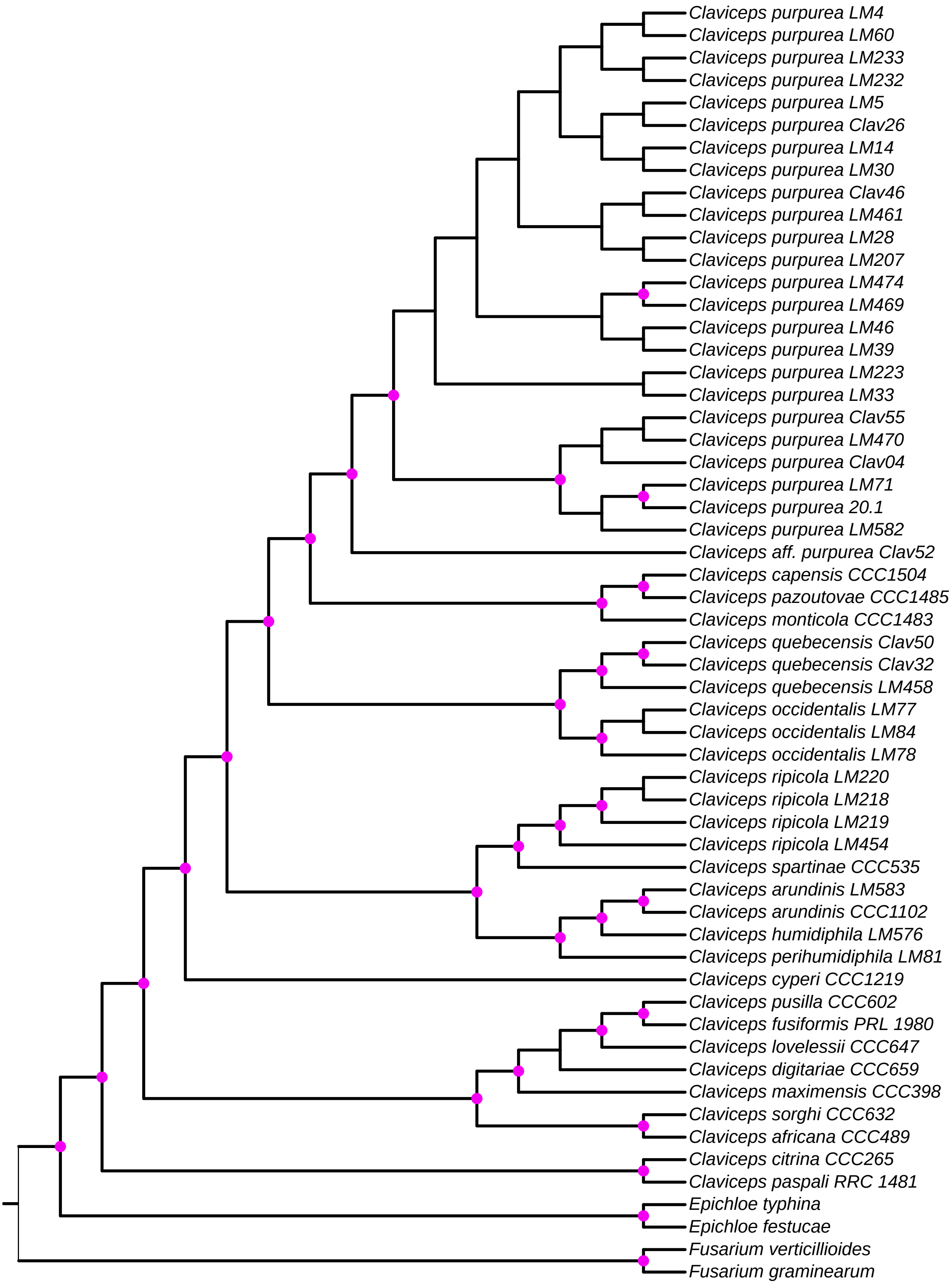

Figure S3: Maximum parsimony phylogenetic reconstruction of the *Claviceps* genus using amino acid sequences of 2,002 single copy orthologs with 1000 bootstrap replicates. Pink dots at branches represent bootstrap values ≥ 95.

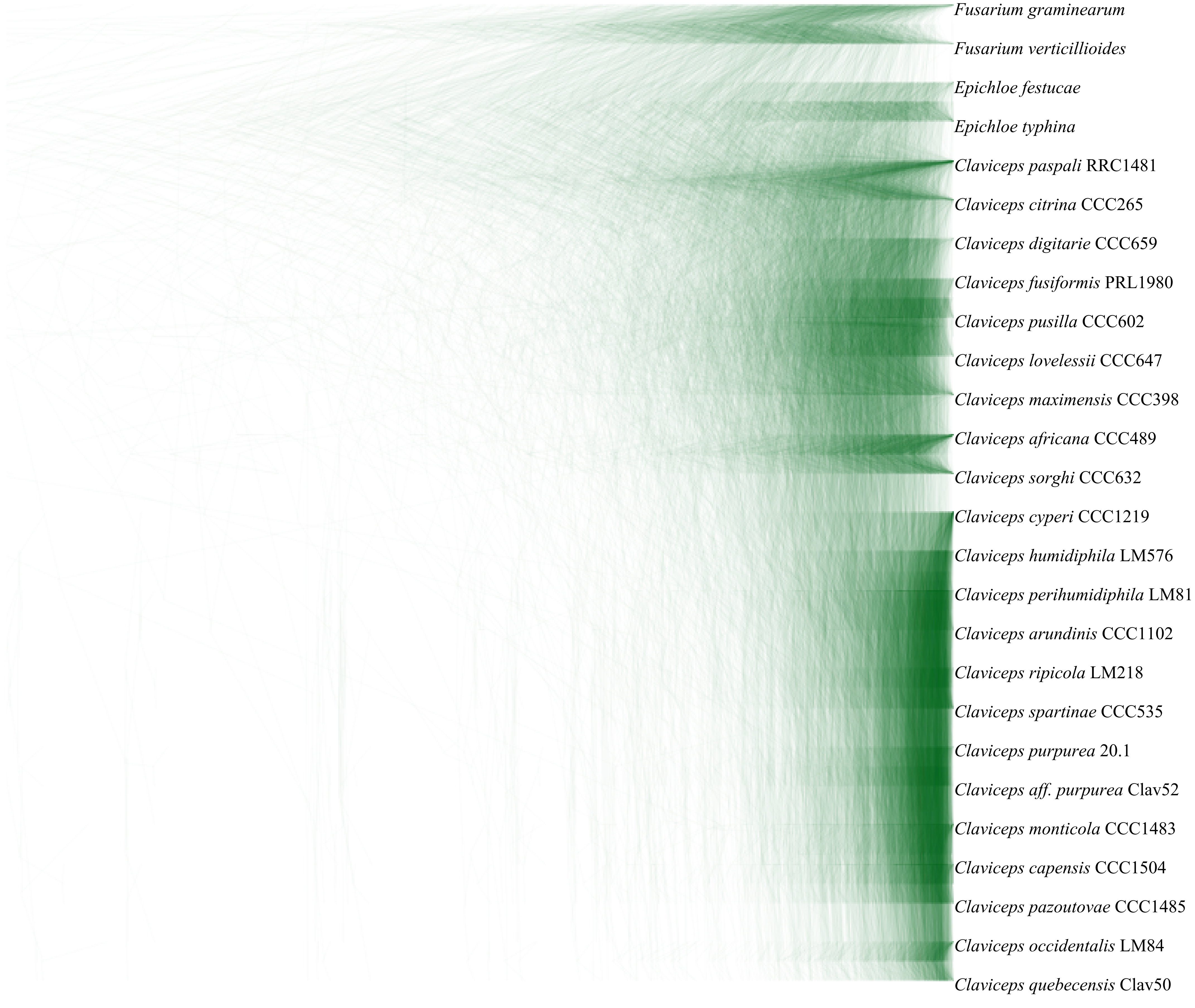

Figure S4: Density consensus tree of 2,002 maximum likelihood phylogenetic reconstructions of the *Claviceps* genus using amino acid sequences of the single-copy orthologs with 1000 bootstrap replicates. Representative isolates from each species were used in this analysis for clarity. Thicker overlapping regions is an indicator of branch support. Tree order was determined by the most frequently occurring tree order.

Figure S5: Phylogenetic reconstructions and genealogy variation of gene trees for the *Claviceps* genus (excluding outgroups). (Line chart) Cumulative distribution of the number of genes per topology. Horizontal dotted lines indicate the half of the genes examined and total genes examined. (Trees) Four most frequent topologies with their corresponding frequencies.

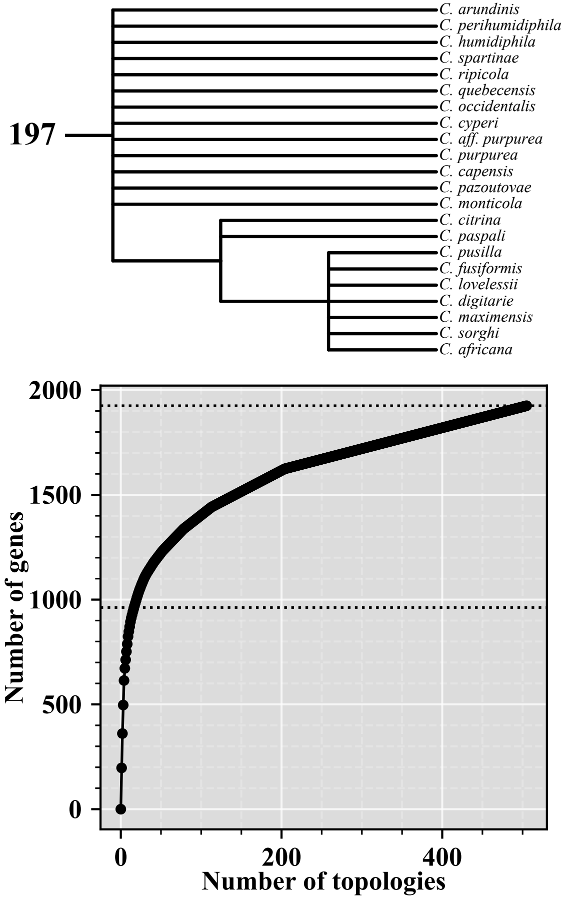

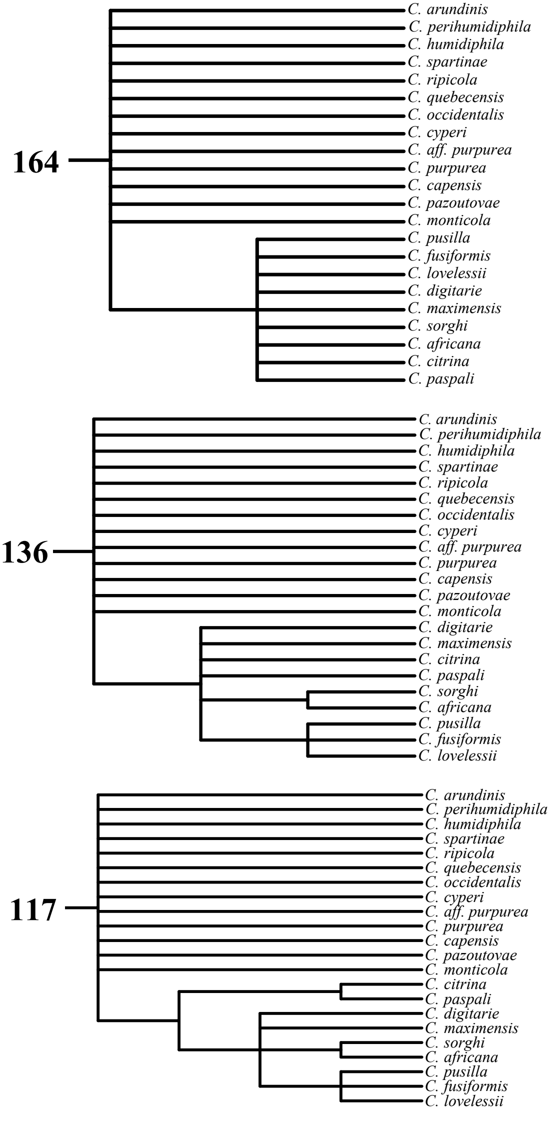

Figure S6: Phylogenetic reconstructions and genealogy variation of gene trees for *Claviceps* section *Claviceps*. (Line chart) Cumulative distribution of the number of genes per topology. Horizontal dotted lines indicate the half of the genes examined and total genes examined. (Trees) six most frequent topologies with their corresponding frequencies.

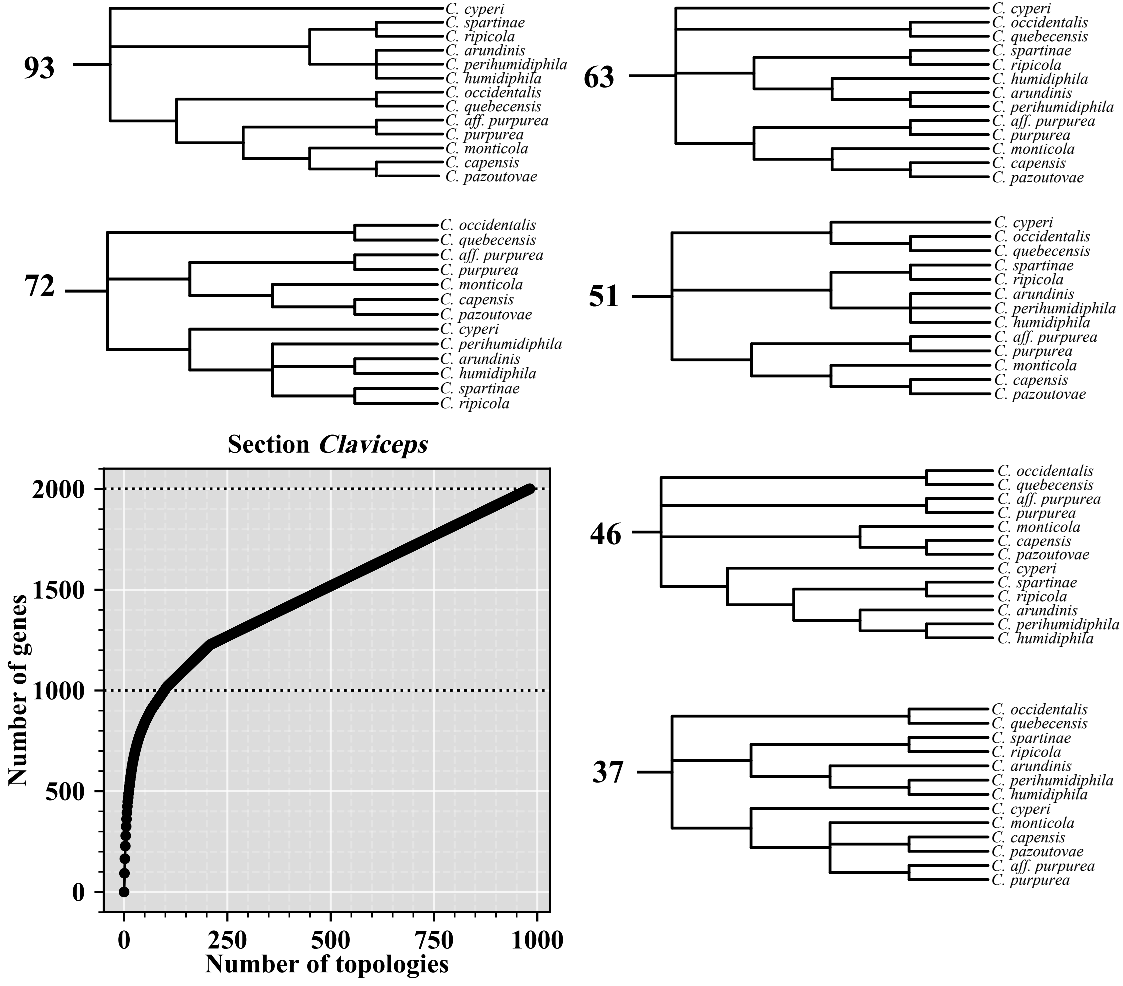

Figure S7: Phylogenetic reconstructions and genealogy variation of gene trees for *Claviceps* section *Pusillae*. (Line chart) Cumulative distribution of the number of genes per topology. Horizontal dotted lines indicate the half of the genes examined and total genes examined. (Trees) six most frequent topologies with their corresponding frequencies.

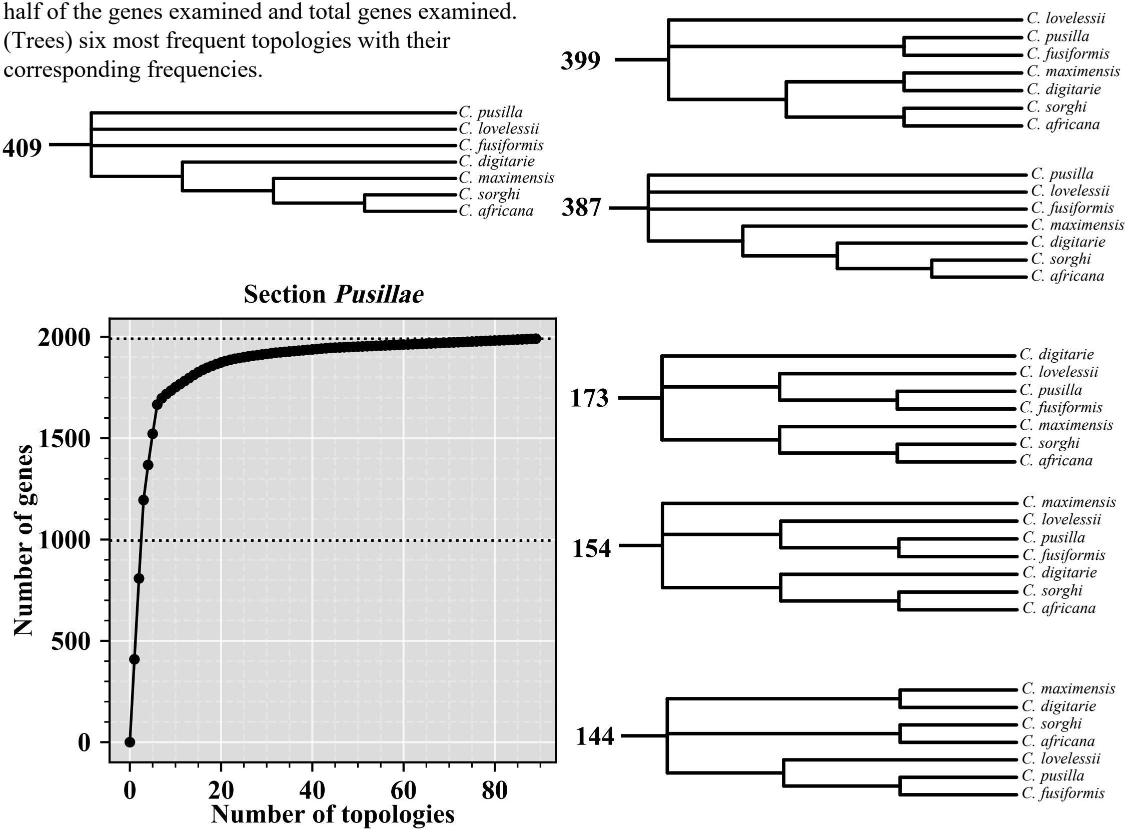

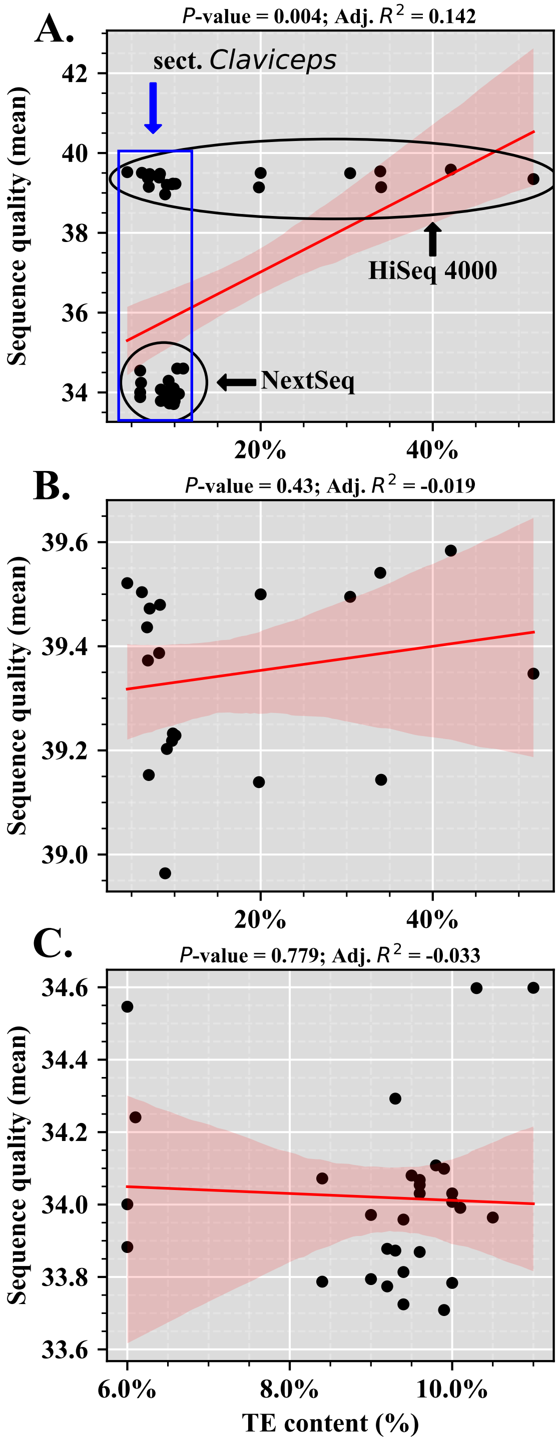

Figure S8: Linear regressions (red lines) of average sequence quality across Illumina raw reads against predicted transposable element (TE) content for each of the 50 newly sequenced *Claviceps* genomes. A.) All genomes. Black ovals indicate different sequencer used and blue rectangle represents sect. *Claviceps* genomes. B.) Genomes sequenced on a HiSeq™ 4000. C.) Genomes sequenced on a NextSeq.

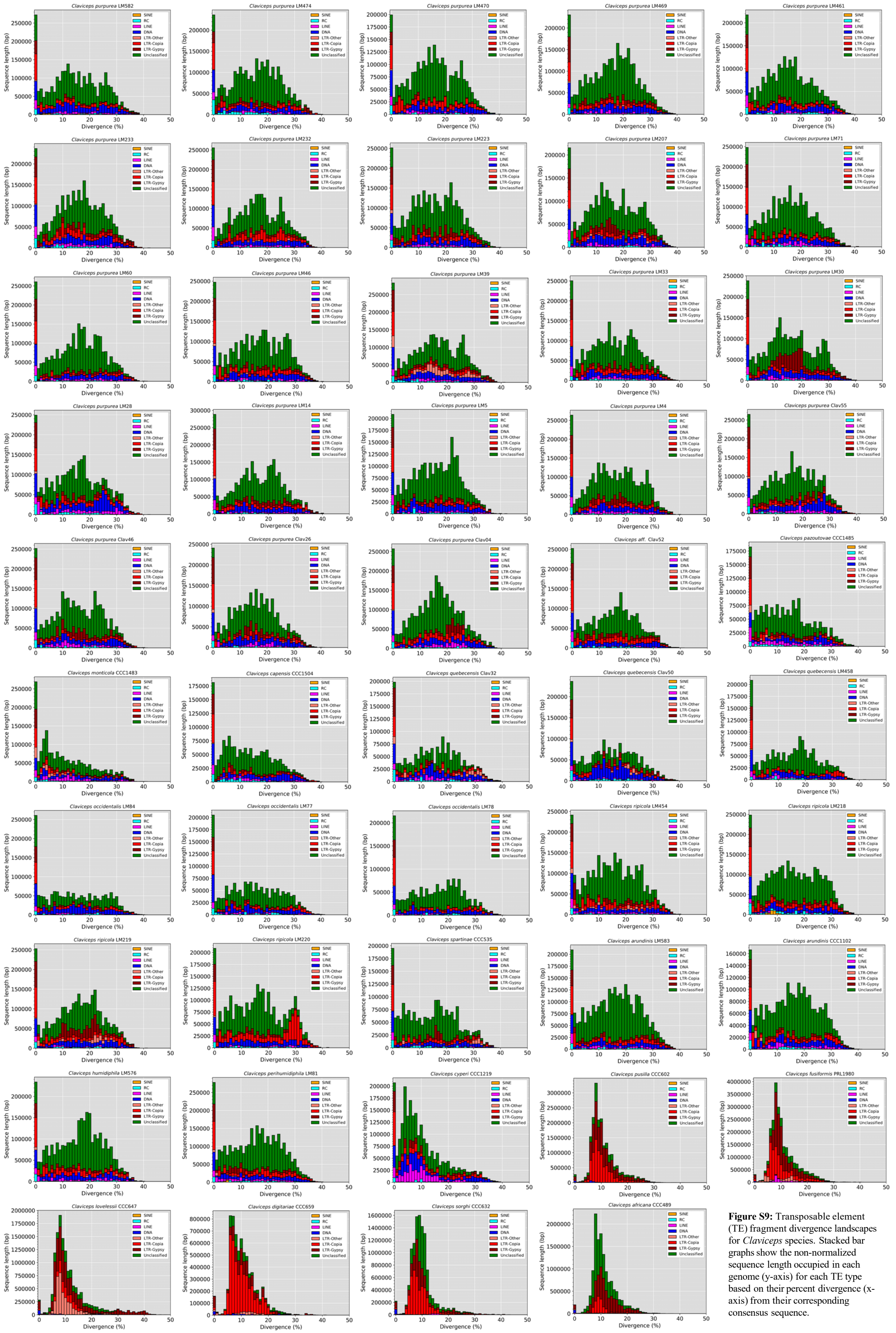

Figure S9: Transposable element (TE) fragment divergence landscapes for *Claviceps* species. Stacked bar graphs show the non-normalized sequence length occupied in each genome (y-axis) for each TE type based on their percent divergence (x-axis) from their corresponding consensus sequence.

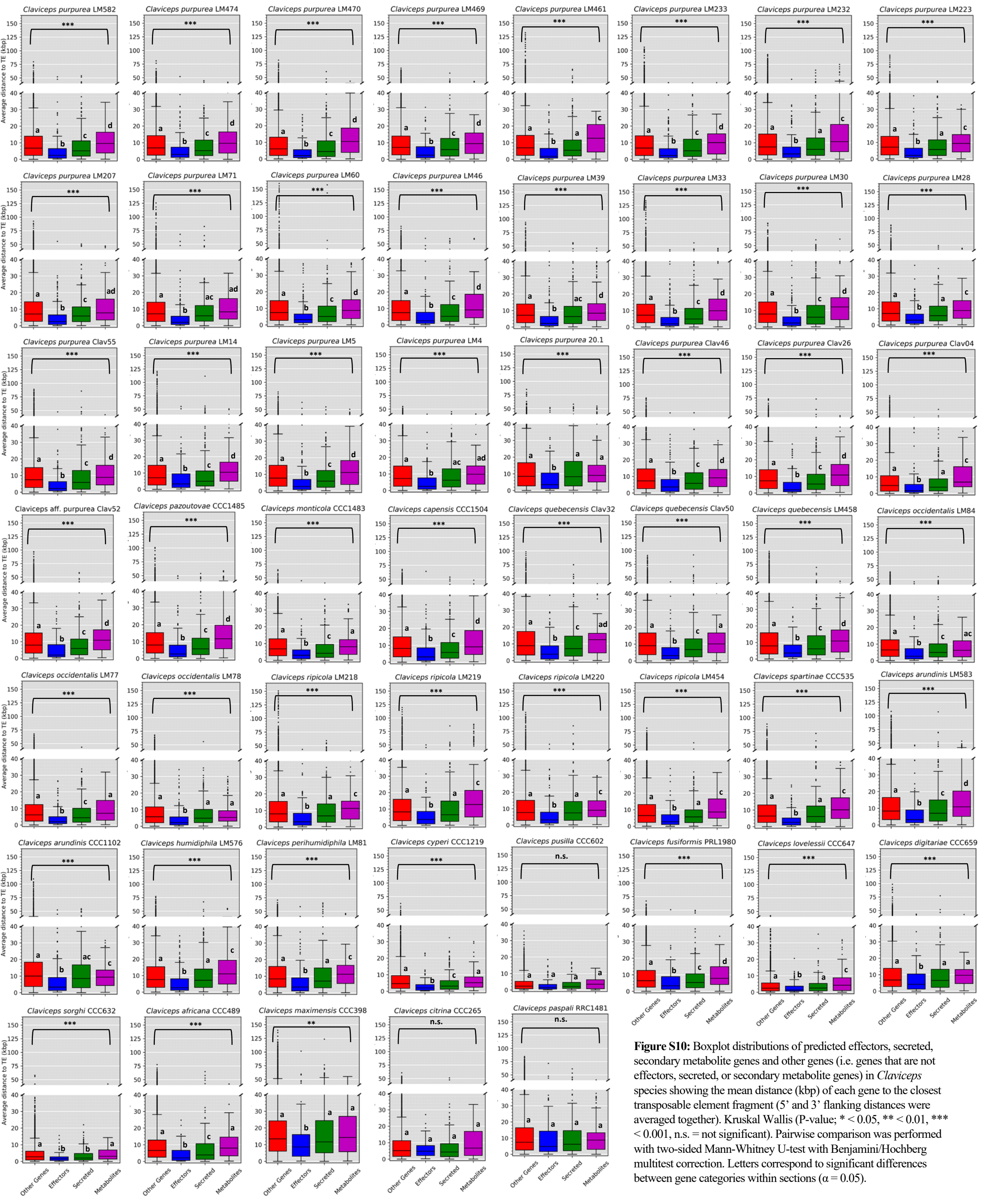

Figure S10: Boxplot distributions of predicted effectors, secreted, secondary metabolite genes and other genes (i.e. genes that are not effectors, secreted, or secondary metabolite genes) in *Claviceps* species showing the mean distance (kbp) of each gene to the closest transposable element fragment (5' and 3' flanking distances were averaged together). Kruskal Wallis (P-value; * < 0.05, ** < 0.01, *** < 0.001, n.s. = not significant). Pairwise comparison was performed with two-sided Mann-Whitney U-test with Benjamini/Hochberg multitest correction. Letters correspond to significant differences between gene categories within sections (α = 0.05).

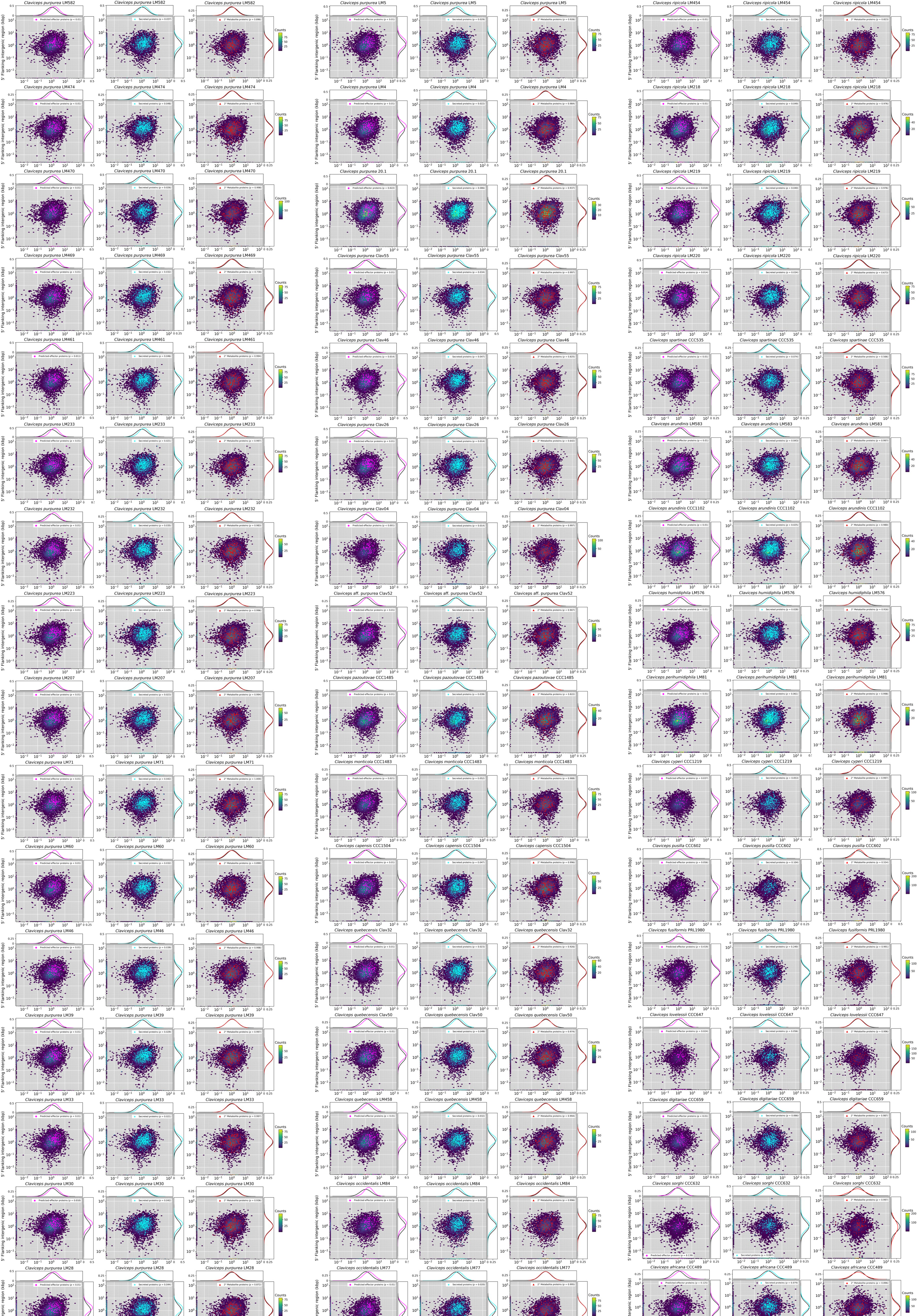

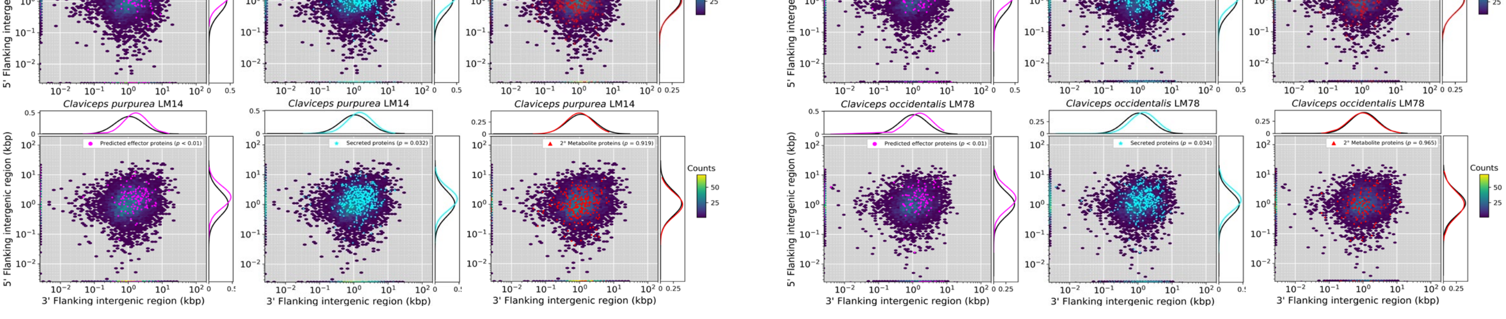

Figure S11: Gene density as a function of flanking 5' and 3' intergenic region size (y and x-axis) of *Claviceps* species. Colored hexbins indicate the intergenic lengths of all genes with color-code indicating the frequency distribution (gene count) according to the legend on the right. Overlaid markers indicate specific gene types corresponding to legends in the top right within each plot. Line graphs (top and right of each plot) depict the frequency distributions of specific gene types (corresponding legend color) and all other genes not of the specific type (black). For visualization purposes the first genes of contig (5' end) are plotted along the x-axis and the last gene of each contig (3' end) are plotted along the y-axis. For information on statistical test see Methods.

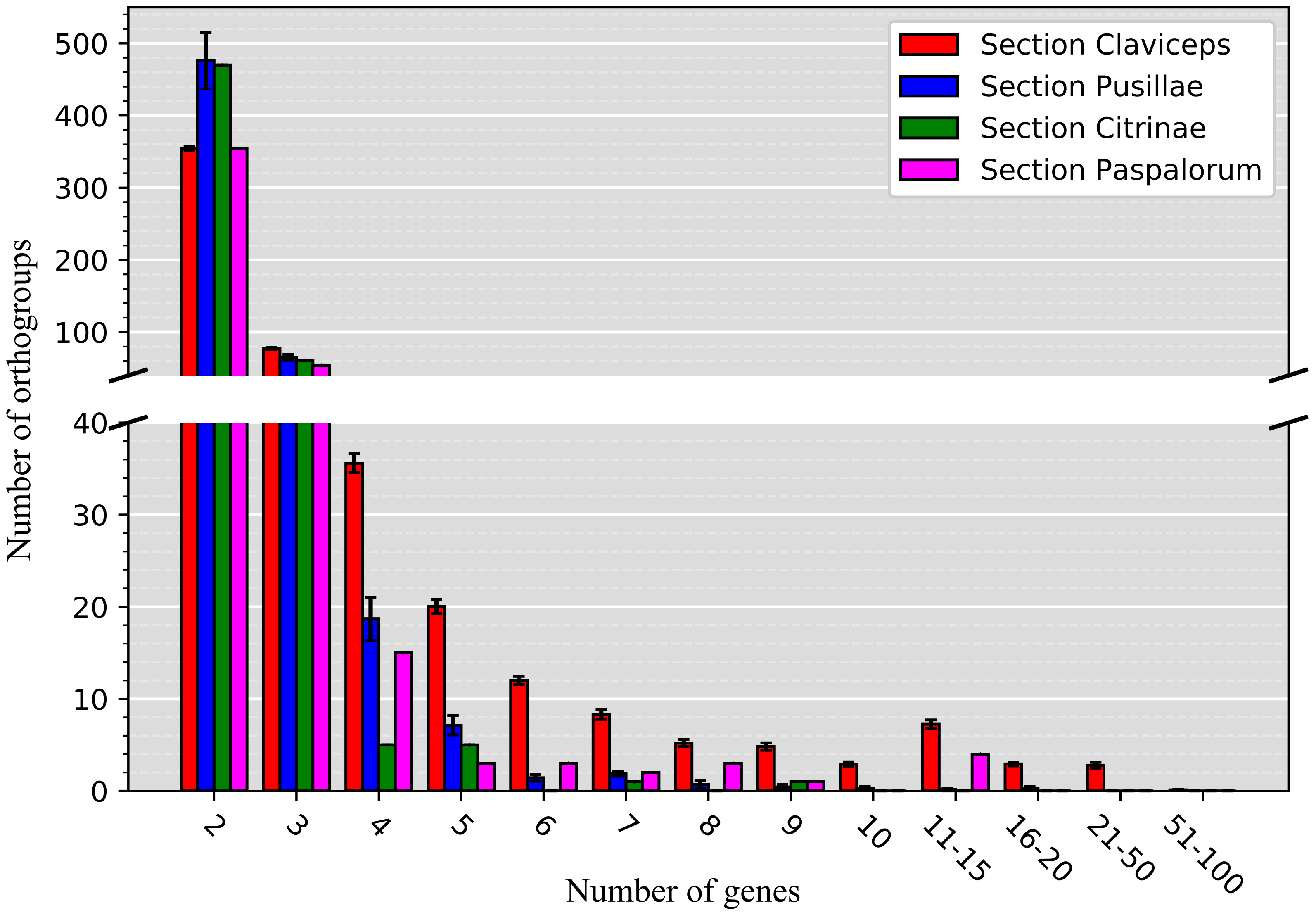

Figure S12: Mean number of orthogroups (y-axis) in each section of the genus *Claviceps* containing X number of genes (x-axis), not including single gene orthogroups for better visualization of paralogs. Bars represent standard error.

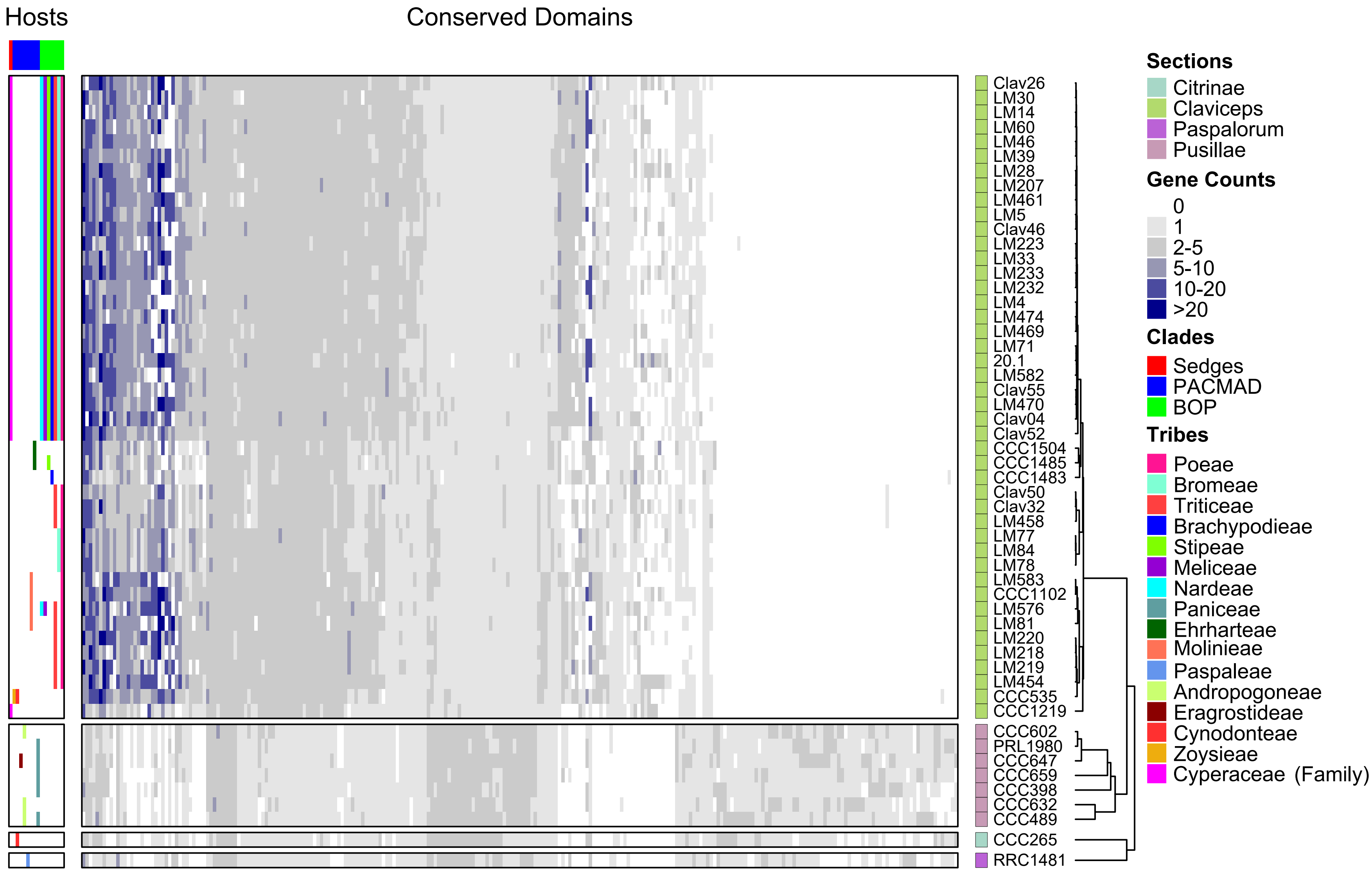

Figure S13: Heatmap of gene counts in the remaining orthogroups containing genes encoding conserved protein domains for all 53 *Claviceps* strains ordered based on ML tree in Fig. 1 and separated by sections. Orthogroups are ordered based on hierarchical clustering. The host spectrum (left) is generalized across species, as no literature has determined the existence of race specific isolates within species, is shown on the left side of the figure determined from literature review of field collected samples (Supplementary Material in Píchová *et al.* 2018) and previous inoculation tests Campbell (1957) and Liu *et al.* (*Accepted*).

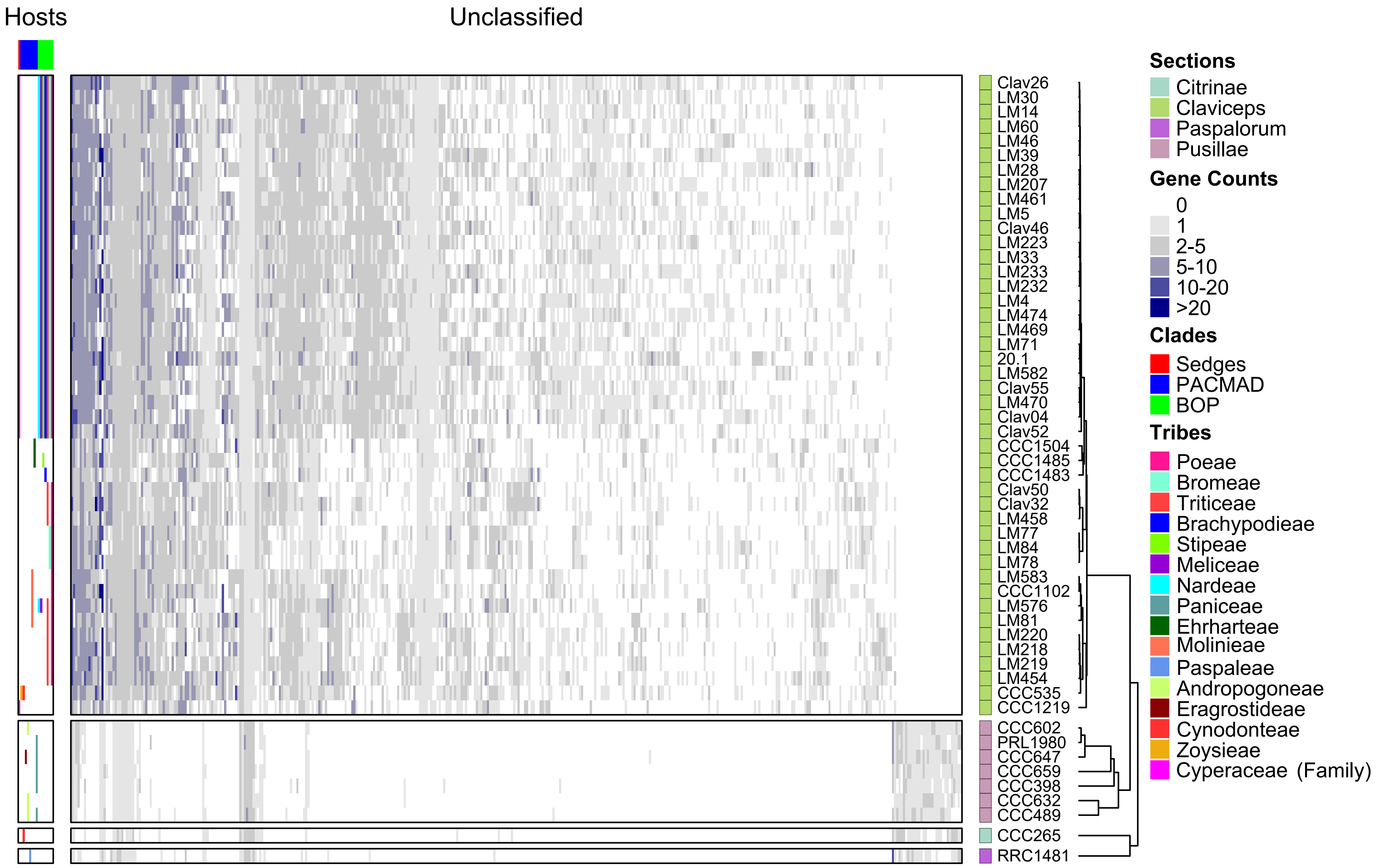

Figure S14: Heatmap of gene counts in remaining orthogroups containing unclassified genes for all 53 *Claviceps* strains ordered based on ML tree in Fig. 1 and separated by sections. Orthogroups are ordered based on hierarchical clustering. The host spectrum (left) is generalized across species, as no literature has determined the existence of race specific isolates within species, is shown on the left side of the figure determined from literature review of field collected samples (Supplementary Material in Píchová *et al.* 2018) and previous inoculation tests Campbell (1957) and Liu *et al.* (*Accepted*).

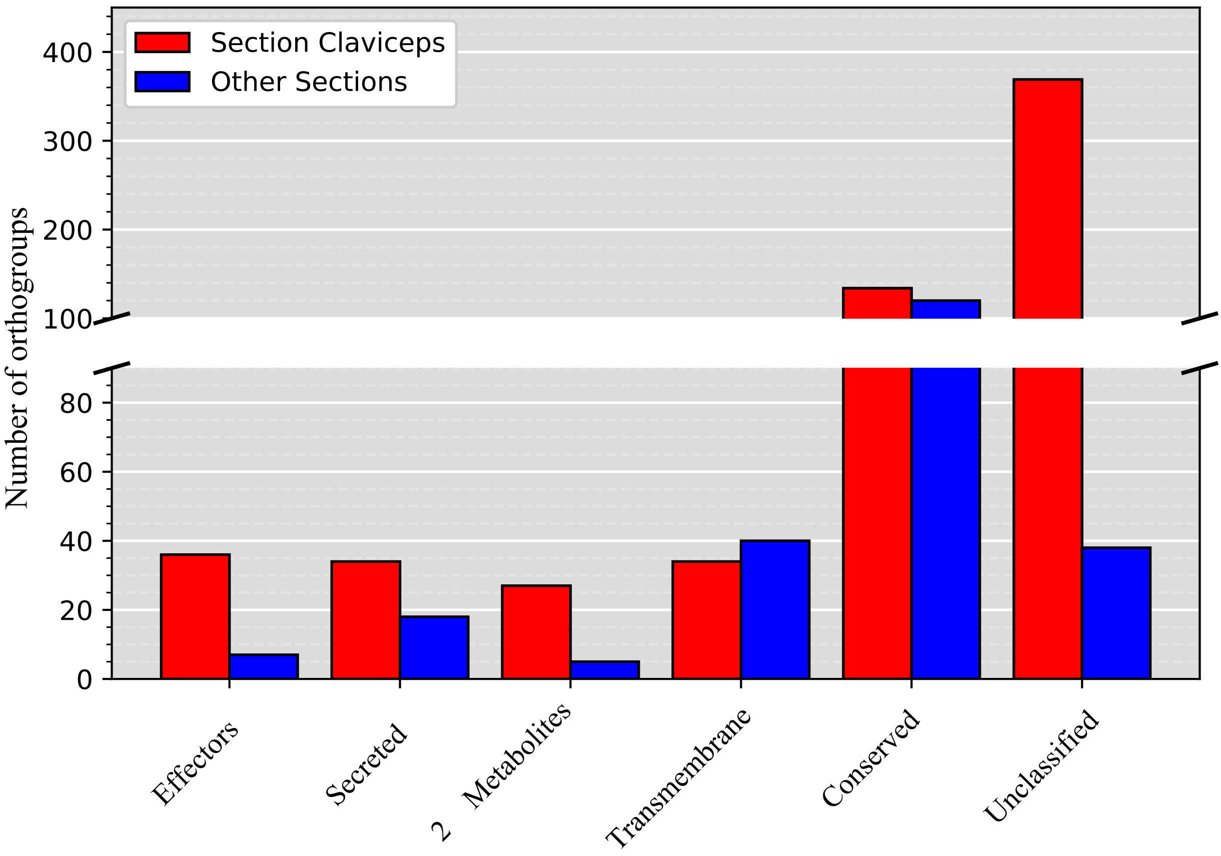

Figure S15: Number of orthogroups showing significantly ($P < 0.01$) greater expansion in respective *Claviceps* sections. Other sections include the combination of sects. *Pusillae*, *Citrinae*, and *Paspalorum*.

Table S1: Collection and accession information for strains used in this study.

Organism	Strain	Strain alias	NCBI Accession	SRA Accession	Culture Collection	Location	Host	Collection Date
References:								
Claviceps purpruea	20.1		SAMEA2272775	--	--	Germany	*Secale cereale*	~1988
Claviceps fusiformis	PRL 1980		SAMN02981339	--	--	Africa: Cote d'Ivoire	*Pennisetum typhoideum*	~1958
Claviceps paspali	RRC 1481		SAMN02981342	--	--	USA: Georgia, Mansfield	*Paspalum* sp.	~2001
This study:								
Claviceps purpruea	Clav04		SAMN11159846	SRR8785178	‡	USA: Colorado, San Luis Valley	*Bromus inermis*	2016
Claviceps purpruea	Clav26		SAMN11159847	SRR8785181	‡	USA: Colorado, San Luis Valley	*Hordeum vulgare*	2016
Claviceps purpruea	Clav46		SAMN11159848	SRR8785180	‡	USA: Wyoming, Worland	*Secale cereale*	2016
Claviceps purpruea	Clav55		SAMN11159850	SRR8785174	‡	New Zealand	*Lolium perenne*	2017
Claviceps purpruea	LM4		SAMN11159851	SRR8785145	DAOMC:250624	Canada: Manitoba	*Tricosecale*	1996
Claviceps purpruea	LM5		SAMN11159852	SRR8785146	DAOMC:250625	Canada: Manitoba	*Hordeum vulgare*	1996
Claviceps purpruea	LM14		SAMN11159853	SRR8785147	DAOMC:250634	Canada: Saskatchewan	*Hordeum vulgare*	1996
Claviceps purpruea	LM28	Jmenzies_81	SAMN11159854	SRR6985966†	DAOMC:250647	Canada: Saskatchewan	*Triticum aestivum*	2000
Claviceps purpruea	LM30		SAMN11159855	SRR8785151	DAOMC:250649	Canada: Saskatchewan	*Secale cereale*	2000
Claviceps purpruea	LM33		SAMN11159856	SRR8785141	DAOMC:250652	Canada: Manitoba	*Secale cereale*	2015
Claviceps purpruea	LM39		SAMN11159857	SRR8785142	DAOMC:250658	Canada: Saskatchewan	*Triticum turgidum subsp. durum*	2000
Claviceps purpruea	LM46		SAMN11159858	SRR8785143	DAOMC:250663	Canada: Alberta	*Triticum turgidum subsp. durum*	2000
Claviceps purpruea	LM60		SAMN11159859	SRR8785144	DAOMC:250680	Canada: Manitoba	*Avena sativa*	2005
Claviceps purpruea	LM71		SAMN11159860	SRR8785148	DAOMC:250720	United Kingdom	*Alopercurus myosuroides*	2004
Claviceps purpruea	LM207		SAMN11159861	SRR8785149	‡	Canada: Manitoba	*Elymus repens*	2014
Claviceps purpruea	LM223		SAMN11159862	SRR8785164	DAOMC:250814	Canada: Manitoba	*Bromus riparius*	2014
Claviceps purpruea	LM232		SAMN11159863	SRR8785161	DAOMC:250822	Canada: Manitoba	*Phalaris canariensis*	2014
Claviceps purpruea	LM233		SAMN11159864	SRR8785162	‡	Canada: Manitoba	*Phalaris canariensis*	2014
Claviceps purpruea	LM461		SAMN11159865	SRR8785163	DAOMC:251847	Canada: Quebec	*Elymus repens*	2016
Claviceps purpruea	LM469		SAMN11159866	SRR8785165	‡	Canada: Ontario	*Triticum aestivum*	2016
Claviceps purpruea	LM470		SAMN11159867	SRR8785166	‡	Canada: Ontario	*Elymus repens*	2016
Claviceps purpruea	LM474		SAMN11159868	SRR8785167	‡	Canada: Ontario	*Hordeum vulgare*	2016
Claviceps purpruea	LM582	CCC771	SAMN11159869	SRR6985962†	DAOMC:251723	Czech Republic: Bezdedice	*Secale cereale*	2003
Claviceps aff. purpruea	Clav52		SAMN11159849	SRR8785175	‡	USA: Washington	*Poa pratensis*	2017
Claviceps quebecensis	Clav32		SAMN11159882	SRR8785176	‡	USA: Montana, Shephard	*Hordeum vulgare*	2016
Claviceps quebecensis	Clav50		SAMN11159881	SRR8785177	‡	USA: Oklahoma, Hoop house Ardmore	*Elymus sp.*	2017
Claviceps quebecensis	LM458		SAMN11159883	SRR6985957†	DAOMC:251898	Canada:Quebec, Cote Nord	*Ammophila* (plant)	2015
Claviceps occidentalis	LM77		SAMN11159879	SRR8785179	DAOMC:250577	Canada: Alberta	*Phleum pratense*	2016
Claviceps occidentalis	LM78		SAMN11159878	SRR6985960†	DAOMC:250578	Canada: Alberta, North Star	*Bromus inermis*	1956
Claviceps occidentalis	LM84		SAMN11159876	SRR8785170	DAOMC:250590	Canada: British Columbia	*Bromus inermis*	2016
Claviceps ripicola	LM218	JM_7.2	SAMN11159875	SRR6985964†	DAOMC:251843	Canada: Manitoba, Grants Field Snowflake	*Phalaris arundinacea*	2014
Claviceps ripicola	LM219		SAMN11159874	SRR8785169	DAOMC:250811	Canada: Manitoba	*Phalaris arundinacea*	2014
Claviceps ripicola	LM220		SAMN11159873	SRR8785168	DAOMC:250812	Canada: Manitoba	*Phalaris arundinacea*	2014
Claviceps ripicola	LM454	139	SAMN11159872	SRR6985963†	DAOMC:251845	Canada: Quebec, MRC Maria-Chapdelaine	*Ammophila breviligulata*	2014
Claviceps spartinae	CCC535		SAMN11159888	SRR8785160	CCC:535	United Kingdom: Marchwood	*Sporobolus anglicus*	1999
Claviceps arundinis	LM583	CCC933	SAMN11159894	SRR6985961†	DAOMC:251724/CCC:933	Czech Republic: Haklovy Dvory, Stary Vrbensky pond	*Phragmites australis*	2008
Claviceps arundinis	CCC1102		SAMN11159893	SRR8785153	CCC:1102	France: D973 Rte de Beaune	*Phragmites australis*	2009
Claviceps humidiphila	LM576	CCC434	SAMN11159871	SRR6985959†	DAOMC:251717/CCC:434	Germany: Bavaria	*Dactylis sp.*	1998
Claviceps perihumidiphila	LM81		SAMN11159877	SRR6985958†	DAOMC:250581	Canada: Alberta, Metiskow	*Elymus albicans*	1956
Claviceps cyperi	CCC1219		SAMN11159895	SRR8785154	CCC:1219	South Africa: Kempton Park	*Cyperus esculentus*	2012
Claviceps capensis	CCC1504		SAMN11159898	SRR8785171	CCC:1504T	South Africa: Cape Town, Western Cape	*Ehrharta villosa*	2014
Claviceps pazoutovae	CCC1485		SAMN11159897	SRR8785152	CCC:1485T	South Africa: Hogsback, Eastern Cape	*Stipa dregeana*	2014
Claviceps monticola	CCC1483		SAMN11159896	SRR8785150	CCC:1483T	South Africa: Hogsback, Eastern Cape	*Brachypodium sp.*	2014
Claviceps pusilla	CCC602		SAMN11159889	SRR8785157	CCC:602	Zimbabwe: Matopos	*Bothriochloa insculpta*	2000
Claviceps lovelessii	CCC647		SAMN11159891	SRR8785155	CCC:647T	Zimbabwe: Matopos, Matopos Research Station	*Eragrostis sp.*	2001
Claviceps digitariae	CCC659		SAMN11159892	SRR8785156	CCC:659	Africa: Botswana	*Digitaria eriantha*	--
Claviceps maximensis	CCC398		SAMN11159886	SRR8785172	CCC:398	Paraguay: Chaco	*Megathyrsus maximus*	1997
Claviceps sorghi	CCC632		SAMN11159890	SRR8785158	CCC:632	India: Karnataka, Jewargi, Gulbarga	*Sorghum bicolor*	2000
Claviceps africana	CCC489		SAMN11159887	SRR8785159	CCC:489	Mexico: Celaya, Guanajuato	*Sorghum bicolor*	1998
Claviceps citrina	CCC265		SAMN11159885	SRR8785173	CCC:265	Mexico: Texcoco (semillero) 6.5km to Mexico City	*Distichlis spicata*	1996

† SRA data first published in Nguyen *et al.* 2018

‡ Cultures available at the lab of Dr. Vamsi Nalam, Colorado State University, Fort Collins, CO or Dr. Miao Liu Ottawa, Research and Development Centre, Agriculture and Agri-Food Canada, Ottawa, Canada

Table S2: Number of genes with functional protein classifications for all 53 *Claviceps* genomes in this study.

Organism	Strain	Section	Protein function							
						Secondary metabolites				
			Conserved domains	MEROP domains	CAZY -mes	Backbone enzymes	Other associated genes	Trans-membrane	Secreted signals	Predicted effectors
**References:**										
C. purpruea	20.1	Claviceps	6560	255	243	36	285	1114	547	199
C. fusiformis	PRL1980	Pusillae	6178	249	244	26	201	1315	521	152
C. paspali	RRC1481	Paspalorum	5943	235	230	23	162	1216	565	196
**This study:**										
C. purpruea	Clav04	Claviceps	6233	246	231	33	166	1145	557	221
C. purpruea	Clav26	Claviceps	6221	263	229	35	220	1158	592	226
C. purpruea	Clav46	Claviceps	6181	266	229	35	246	1151	560	198
C. purpruea	Clav55	Claviceps	6113	260	231	33	220	1166	553	195
C. purpruea	LM4	Claviceps	6132	249	233	36	194	1149	584	231
C. purpruea	LM5	Claviceps	6179	257	231	36	251	1154	547	193
C. purpruea	LM14	Claviceps	6132	259	227	35	238	1164	529	172
C. purpruea	LM28	Claviceps	6260	254	233	32	247	1156	540	188
C. purpruea	LM30	Claviceps	6167	262	229	38	230	1156	588	220
C. purpruea	LM33	Claviceps	6193	263	226	36	228	1143	597	237
C. purpruea	LM39	Claviceps	6156	265	232	32	224	1150	584	228
C. purpruea	LM46	Claviceps	6125	261	225	36	214	1158	554	201
C. purpruea	LM60	Claviceps	6126	256	231	37	253	1147	553	195
C. purpruea	LM71	Claviceps	6095	240	221	36	212	1152	550	219
C. purpruea	LM207	Claviceps	6120	259	220	33	218	1140	571	224
C. purpruea	LM223	Claviceps	6076	234	230	34	216	1146	564	214
C. purpruea	LM232	Claviceps	6169	260	232	35	249	1156	555	199
C. purpruea	LM233	Claviceps	6294	264	230	34	232	1162	570	207
C. purpruea	LM461	Claviceps	6223	237	231	34	212	1154	550	210
C. purpruea	LM469	Claviceps	6106	255	229	36	237	1148	542	185
C. purpruea	LM470	Claviceps	6195	256	223	32	178	1144	533	184
C. purpruea	LM474	Claviceps	6118	242	222	36	254	1162	522	191
C. purpruea	LM582	Claviceps	6132	257	226	32	197	1141	545	198
C. aff. purpruea	Clav52	Claviceps	6078	252	225	33	217	1153	523	177
C. quebecensis	Clav32	Claviceps	6057	244	226	31	235	1157	522	174
C. quebecensis	Clav50	Claviceps	5986	248	228	32	228	1139	524	174
C. quebecensis	LM458	Claviceps	6007	243	226	28	207	1135	508	154
C. occidentalis	LM77	Claviceps	6020	243	222	34	152	1132	517	182
C. occidentalis	LM78	Claviceps	6052	246	223	34	129	1133	513	174
C. occidentalis	LM84	Claviceps	6088	244	223	33	156	1129	517	180
C. ripicola	LM218	Claviceps	6090	249	228	32	223	1136	545	203
C. ripicola	LM219	Claviceps	6133	255	226	32	238	1130	538	188
C. ripicola	LM220	Claviceps	6168	249	225	33	222	1122	564	211
C. ripicola	LM454	Claviceps	6163	261	224	33	228	1134	554	210
C. spartinae	CCC535	Claviceps	6146	260	227	37	212	1153	504	163
C. arundinis	LM583	Claviceps	6102	253	225	35	252	1140	526	186
C. arundinis	CCC1102	Claviceps	6213	259	226	34	254	1130	548	195
C. humidiphila	LM576	Claviceps	6147	253	227	32	250	1152	564	211
C. perihumidiphila	LM81	Claviceps	6106	238	225	29	241	1124	522	176
C. cyperi	CCC1219	Claviceps	5774	228	202	25	110	1072	392	97
C. capensis	CCC1504	Claviceps	5989	245	225	33	217	1145	475	137
C. pazoutovae	CCC1485	Claviceps	5954	232	223	30	195	1137	478	150
C. monticola	CCC1483	Claviceps	5908	248	220	30	177	1118	481	148
C. pusilla	CCC602	Pusillae	6276	252	227	25	51	1235	461	138
C. lovelessii	CCC647	Pusillae	6351	248	228	26	84	1230	525	174
C. digitariae	CCC659	Pusillae	6195	254	239	25	162	1213	513	158
C. maximensis	CCC398	Pusillae	6100	244	235	23	203	1172	468	126
C. sorghi	CCC632	Pusillae	6085	241	222	31	86	1139	425	123
C. africana	CCC489	Pusillae	6057	241	221	30	135	1151	471	145
C. citrina	CCC265	Citrinae	5879	224	207	27	93	1100	368	85

Table S3: Additional annotated genomes used in OrthoFinder analysis for finding orthologous gene clusters (orthogroups).

Organism	Strain	Accession
Acremonium chrysogenum	ATCC 11550	SAMN02799700
Atkinsonella hypoxylon	B4728	http://www.endophyte.uky.edu/
Atkinsonella texensis	B6155	http://www.endophyte.uky.edu/
Balansia obtecta	B249	http://www.endophyte.uky.edu/
Clonostachys rosea	CBS125111	JGI:1032557
Epichloe amarillians	ATCC 200744	http://www.endophyte.uky.edu/
Epichloe aotearoea	ATCC MYA-1229	http://www.endophyte.uky.edu/
Epichloe baconii	ATCC 200745	http://www.endophyte.uky.edu/
Epichloe brachyelytri	E4804	http://www.endophyte.uky.edu/
Epichloe bromicola	AL0426/2	http://www.endophyte.uky.edu/
Epichloe coenophiala	e4163	http://www.endophyte.uky.edu/
Epichloe elymi	ATCC 201551	http://www.endophyte.uky.edu/
Epichloe festucae	Fl1	http://www.endophyte.uky.edu/
Epichloe gansuensis	CDM-2007b	http://www.endophyte.uky.edu/
Epichloe glyceriae	ATCC 200747	http://www.endophyte.uky.edu/
Epichloe inebrians	ATCC MYA-1228	http://www.endophyte.uky.edu/
Epichloe mollis	AL9924	http://www.endophyte.uky.edu/
Epichloe sylvatica	GR 10156	http://www.endophyte.uky.edu/
Epichloe typhina	ATCC 200736	http://www.endophyte.uky.edu/
Epichloe uncinata	CBS 102646	http://www.endophyte.uky.edu/
Fusarium ambrosium	NRRL 20438	SAMN07200640
Fusarium avenaceum	Fave_LH27	SAMN02850900
Fusarium fujikuroi		SAMEA4440726
Fusarium fujikuroi	B14	SAMEA4436914
Fusarium fujikuroi	C1995	SAMEA4440729
Fusarium fujikuroi	E282	SAMEA4440730
Fusarium fujikuroi	FGSC8932	SAMN03075939
Fusarium fujikuroi	FSU48	SAMEA4440731
Fusarium fujikuroi	IMI58289	SAMEA3724789
Fusarium fujikuroi	KSU3368	SAMN03075941
Fusarium fujikuroi	KSUX10626	SAMN03075940
Fusarium fujikuroi	m657	SAMEA4440732
Fusarium fujikuroi	MRC2276	SAMEA4440733
Fusarium fujikuroi	NCIM1100	SAMEA4440734
Fusarium graminearum	PH-1/NRRL 31084	SAMN02953593
Fusarium kuroshium	AF-12	SAMN07200645
Fusarium langsethiae	Fl201059	SAMN03274931
Fusarium longipes	NRRL 20695	SAMN08631279
Fusarium mangiferae	MRC7560	SAMEA3862491
Fusarium oxysporum f. sp. cepae	FoCFus2	SAMN05529097
Fusarium oxysporum f. sp. conglutinans	54008	SAMN02981380
Fusarium oxysporum f. sp. cubense	54006	SAMN02981379
Fusarium oxysporum f. sp. lycopersici	4287	SAMN02953675
Fusarium oxysporum f. sp. melonis	26406	SAMN02981378
Fusarium oxysporum f. sp. narcissi	N139	SAMN05526391
Fusarium oxysporum f. sp. pisi	HDV247	SAMN02981366
Fusarium oxysporum f. sp. radicis-cucumerinum	Forc016	SAMN04348764
Fusarium oxysporum f. sp. raphani	54005	SAMN02981381
Fusarium oxysporum f. sp. vasinfectum	25433	SAMN02981377
Fusarium poae	2516	SAMN05178635
Fusarium proliferatum	ET1	SAMEA3862493
Fusarium pseudograminearum	CS3096	SAMN02981337
Fusarium solani (Nectria haematococca)	77-13-4	SAMN02746079
Fusarium sporotrichioides	NRRL 3299	SAMN08631227
Fusarium venenatum	A3/5	SAMEA2827224
Fusarium verticillioides	7600	SAMN02953630
Periglandula ipomoeae	IasaF13	http://www.endophyte.uky.edu/
Purpureocillium lilacinum	PLFJ-1	SAMN04404347
Saccharomyces cerevisiae	S288C	PRJNA43747
Stachybotrys chlorohalonata	IBT 40285	SAMN01819006
Trichoderma arundinaceum	IBT 40837	SAMN06320351
Trichoderma asperellum	CBS 433.97	SAMN00769595
Trichoderma atroviride	IMI 206040	SAMN02744066
Trichoderma citrinoviride	TUCIM 6016	SAMN05369575
Trichoderma gamsii	T6085	SAMN02849381
Trichoderma guizhouense	NJAU 4742	SAMN04535176
Trichoderma harzianum	CBS22695	SAMN00761861
Trichoderma harzianum	T6776	SAMN02851310
Trichoderma harzianum	Tr1	SAMN06219536
Trichoderma harzianum	TR274	SAMN07456232
Trichoderma harzianum	M10 v1	JGI:1185309
Trichoderma harzianum	T22 v1	JGI:1185313
Trichoderma longibrachiatum	ATCC 18648	SAMN00767620
Trichoderma parareesei	CBS 125925	SAMN03784587
Trichoderma reesei	QM6a	SAMN02746107
Trichoderma virens	Gv29-8	SAMN02744059
Ustilaginoidea virens	UV-8b	SAMN02693461
Ustilago maydis	521	SAMN02900459

Table S4: Number and percent of that appeared on a contig alone and were removed from gene density compartmentalization analysis.

Species	Strain	Section	All genes	Genes removed (# [%])	Predicted effector genes	Predicted effector genes removed (# [%])	Secreted genes	Secreted genes removed (# [%])	Secondary Metabolite genes	Secondary Metabolite genes removed (# [%]))
C. citrina	CCC265	Citrinae	7329	492 [6.71%]	85	7 [8.24%]	368	27 [7.34%]	120	3 [2.5%]
C. arundinis	CCC1102	Claviceps	8320	166 [2%]	195	6 [3.08%]	548	7 [1.28%]	288	4 [1.39%]
C. cyperi	CCC1219	Claviceps	7244	429 [5.92%]	97	19 [19.59%]	392	37 [9.44%]	135	2 [1.48%]
C. monticola	CCC1483	Claviceps	7696	245 [3.18%]	148	5 [3.38%]	481	25 [5.2%]	207	1 [0.48%]
C. pazoutovae	CCC1485	Claviceps	7754	223 [2.88%]	150	9 [6%]	478	22 [4.6%]	225	1 [0.44%]
C. capensis	CCC1504	Claviceps	7811	226 [2.89%]	137	6 [4.38%]	475	13 [2.74%]	250	2 [0.8%]
C. spartinae	CCC535	Claviceps	8121	312 [3.84%]	163	7 [4.29%]	504	32 [6.35%]	249	4 [1.61%]
C. purpurea	Clav04	Claviceps	7965	859 [10.78%]	221	47 [21.27%]	557	70 [12.57%]	199	6 [3.02%]
C. purpurea	Clav26	Claviceps	8500	237 [2.79%]	226	9 [3.98%]	592	12 [2.03%]	255	2 [0.78%]
C. quebecensis	Clav32	Claviceps	8058	174 [2.16%]	174	7 [4.02%]	522	11 [2.11%]	266	0 [0%]
C. purpurea	Clav46	Claviceps	8327	270 [3.24%]	198	6 [3.03%]	560	16 [2.86%]	281	1 [0.36%]
C. quebecensis	Clav50	Claviceps	7846	200 [2.55%]	174	5 [2.87%]	524	20 [3.82%]	260	0 [0%]
C. aff. purpurea	Clav52	Claviceps	8091	225 [2.78%]	177	7 [3.95%]	523	17 [3.25%]	250	3 [1.2%]
C. purpurea	Clav55	Claviceps	8173	307 [3.76%]	195	17 [8.72%]	553	32 [5.79%]	253	4 [1.58%]
C. purpurea	20.1	Claviceps	8665	38 [0.44%]	199	0 [0%]	547	0 [0%]	321	0 [0%]
C. purpurea	LM14	Claviceps	8191	231 [2.82%]	172	6 [3.49%]	529	10 [1.89%]	273	0 [0%]
C. purpurea	LM207	Claviceps	8230	245 [2.98%]	224	11 [4.91%]	571	15 [2.63%]	251	1 [0.4%]
C. ripicola	LM218	Claviceps	8135	192 [2.36%]	203	8 [3.94%]	545	16 [2.94%]	255	3 [1.18%]
C. ripicola	LM219	Claviceps	8150	231 [2.83%]	188	5 [2.66%]	538	15 [2.79%]	270	0 [0%]
C. ripicola	LM220	Claviceps	8231	218 [2.65%]	211	6 [2.84%]	564	9 [1.6%]	255	2 [0.78%]
C. purpurea	LM223	Claviceps	8239	199 [2.42%]	214	4 [1.87%]	564	9 [1.6%]	250	1 [0.4%]
C. purpurea	LM232	Claviceps	8276	236 [2.85%]	199	10 [5.03%]	555	17 [3.06%]	284	1 [0.35%]
C. purpurea	LM233	Claviceps	8482	235 [2.77%]	207	6 [2.9%]	570	14 [2.46%]	266	0 [0%]
C. purpurea	LM28	Claviceps	8457	256 [3.03%]	188	8 [4.26%]	540	13 [2.41%]	279	1 [0.36%]
C. purpurea	LM30	Claviceps	8310	216 [2.6%]	220	12 [5.45%]	588	17 [2.89%]	268	4 [1.49%]
C. purpurea	LM33	Claviceps	8274	283 [3.42%]	237	8 [3.38%]	597	20 [3.35%]	264	5 [1.89%]
C. purpurea	LM39	Claviceps	8358	233 [2.79%]	228	8 [3.51%]	584	13 [2.23%]	256	0 [0%]
C. purpurea	LM4	Claviceps	8229	241 [2.93%]	231	14 [6.06%]	584	22 [3.77%]	230	1 [0.43%]
C. ripicola	LM454	Claviceps	8252	310 [3.76%]	210	5 [2.38%]	554	10 [1.81%]	261	2 [0.77%]
C. quebecensis	LM458	Claviceps	7857	198 [2.52%]	154	6 [3.9%]	508	20 [3.94%]	235	0 [0%]
C. purpurea	LM46	Claviceps	8228	227 [2.76%]	201	10 [4.98%]	554	15 [2.71%]	250	3 [1.2%]
C. purpurea	LM461	Claviceps	8378	278 [3.32%]	210	18 [8.57%]	550	24 [4.36%]	246	2 [0.81%]
C. purpurea	LM469	Claviceps	8176	218 [2.67%]	185	7 [3.78%]	542	12 [2.21%]	273	0 [0%]
C. purpurea	LM470	Claviceps	8237	354 [4.3%]	184	19 [10.33%]	533	32 [6%]	210	2 [0.95%]
C. purpurea	LM474	Claviceps	8255	245 [2.97%]	191	10 [5.24%]	522	12 [2.3%]	290	2 [0.69%]
C. purpurea	LM5	Claviceps	8284	224 [2.7%]	193	6 [3.11%]	547	14 [2.56%]	287	4 [1.39%]
C. humidiphila	LM576	Claviceps	8236	204 [2.48%]	211	5 [2.37%]	564	10 [1.77%]	282	0 [0%]
C. purpurea	LM582	Claviceps	8193	325 [3.97%]	198	21 [10.61%]	545	34 [6.24%]	229	0 [0%]
C. arundinis	LM583	Claviceps	8083	152 [1.88%]	186	6 [3.23%]	526	11 [2.09%]	287	1 [0.35%]
C. purpurea	LM60	Claviceps	8279	219 [2.65%]	195	6 [3.08%]	553	12 [2.17%]	290	5 [1.72%]
C. purpurea	LM71	Claviceps	8221	251 [3.05%]	219	10 [4.57%]	550	17 [3.09%]	248	1 [0.4%]
C. occidentalis	LM77	Claviceps	7803	359 [4.6%]	182	13 [7.14%]	517	27 [5.22%]	186	5 [2.69%]
C. occidentalis	LM78	Claviceps	7909	322 [4.07%]	174	9 [5.17%]	513	19 [3.7%]	163	6 [3.68%]
C. perihumidiphila	LM81	Claviceps	8130	161 [1.98%]	176	4 [2.27%]	522	6 [1.15%]	270	0 [0%]
C. occidentalis	LM84	Claviceps	7997	224 [2.8%]	180	8 [4.44%]	517	22 [4.26%]	189	3 [1.59%]
C. paspali	RRC 1481	Paspalorum	8145	255 [3.13%]	196	9 [4.59%]	565	27 [4.78%]	185	1 [0.54%]
C. maximensis	CCC398	Pusillae	7893	50 [0.63%]	126	3 [2.38%]	468	4 [0.85%]	226	0 [0%]
C. africana	CCC489	Pusillae	7856	263 [3.35%]	145	3 [2.07%]	471	11 [2.34%]	165	2 [1.21%]
C. pusilla	CCC602	Pusillae	7312	1423 [19.46%]	138	34 [24.64%]	461	98 [21.26%]	76	10 [13.16%]
C. sorghi	CCC632	Pusillae	7171	1037 [14.46%]	123	18 [14.63%]	425	69 [16.24%]	117	5 [4.27%]
C. lovelessii	CCC647	Pusillae	7421	1441 [19.42%]	174	25 [14.37%]	525	89 [16.95%]	110	5 [4.55%]
C. digitariae	CCC659	Pusillae	8032	253 [3.15%]	158	5 [3.16%]	513	21 [4.09%]	187	1 [0.53%]
C. fusiformis	PRL 1980	Pusillae	9046	258 [2.85%]	152	12 [7.89%]	521	29 [5.57%]	227	0 [0%]

Table S5: *P*-values for genomic fluidity differences from two-sample two-sided z-test, bold numbers indicate significance.

	Fusarium-Genus	Epichloe-Genus	Trichoderma-Genus	T.harazium-Species	Pusillae-Section	Claviceps-Genus	F.oxysporum-Species	Claviceps-Section	C.purpurea-Species	F.fujikuroi-Species
Fusarium-Genus	-	8.74E-01	3.78E-01	**3.30E-04**	**1.54E-03**	**7.98E-05**	**4.01E-07**	**4.35E-21**	**6.44E-27**	**3.65E-25**
Epichloe-Genus	-	-	4.94E-01	**2.68E-03**	**6.47E-03**	**7.59E-04**	**4.39E-05**	**1.81E-14**	**2.04E-18**	**9.37E-18**
Trichoderma-Genus	-	-	-	5.29E-02	7.23E-02	**2.01E-02**	**6.26E-03**	**5.33E-09**	**1.12E-11**	**1.73E-11**
T.harazium-Species	-	-	-	-	9.63E-01	5.21E-01	3.35E-01	**2.04E-10**	**8.00E-16**	**2.65E-14**
Pusillae-Section	-	-	-	-	-	6.13E-01	4.96E-01	**3.34E-06**	**3.91E-09**	**7.56E-09**
Claviceps-Genus	-	-	-	-	-	-	9.56E-01	**2.98E-06**	**8.88E-10**	**2.95E-09**
F.oxysporum-Species	-	-	-	-	-	-	-	**3.42E-30**	**3.32E-53**	**7.78E-31**
Claviceps-Section	-	-	-	-	-	-	-	-	**2.05E-11**	**1.35E-04**
C.purpurea-Species	-	-	-	-	-	-	-	-	-	6.25E-01
F.fujikuroi-Species	-	-	-	-	-	-	-	-	-	-

Table S6: Number of duplicated genes and unique gene pairs with a pairwise identity ≥ 80% and the proportion of these gene pairs that are located next to each other (separated by 0 genes) and separated by five or fewer genes (≤ 5 genes) for all 53 Claviceps genomes.

Species	Strain	Section	Gene pairs[†] (#)	Duplicated genes (#)	Separation 0 genes	Separation ≤ 5 genes
C. purpurea	20.1	Claviceps	997	846	11.74	30.19
C. purpurea	Clav04	Claviceps	578	710	8.65	11.94
C. purpurea	Clav26	Claviceps	429	587	17.48	30.77
C. purpurea	Clav46	Claviceps	415	553	18.55	29.64
C. purpurea	Clav55	Claviceps	373	523	16.09	26.81
C. purpurea	LM4	Claviceps	426	591	19.95	34.51
C. purpurea	LM5	Claviceps	412	536	15.78	30.83
C. purpurea	LM14	Claviceps	352	493	19.6	32.95
C. purpurea	LM28	Claviceps	404	542	14.36	25.99
C. purpurea	LM30	Claviceps	352	511	21.88	38.35
C. purpurea	LM33	Claviceps	395	528	18.23	32.66
C. purpurea	LM39	Claviceps	393	521	17.3	28.24
C. purpurea	LM46	Claviceps	383	550	15.4	29.24
C. purpurea	LM60	Claviceps	374	519	21.39	34.22
C. purpurea	LM71	Claviceps	332	484	17.77	31.02
C. purpurea	LM207	Claviceps	354	515	17.8	27.68
C. purpurea	LM223	Claviceps	348	487	21.84	34.2
C. purpurea	LM232	Claviceps	424	542	15.33	26.42
C. purpurea	LM233	Claviceps	673	616	11.59	24.37
C. purpurea	LM461	Claviceps	401	557	14.96	27.93
C. purpurea	LM469	Claviceps	361	489	20.5	32.96
C. purpurea	LM470	Claviceps	410	410	16.34	27.8
C. purpurea	LM474	Claviceps	319	496	18.81	30.72
C. purpurea	LM582	Claviceps	386	512	13.99	24.09
C. aff. purpurea	Clav52	Claviceps	235	355	20.0	31.06
C. capensis	CCC1504	Claviceps	144	247	13.89	21.53
C. pazoutovae	CCC1485	Claviceps	182	270	14.29	20.33
C. monticola	CCC1483	Claviceps	174	272	13.22	21.84
C. occidentalis	LM78	Claviceps	173	278	18.5	26.59
C. occidentalis	LM77	Claviceps	151	250	17.88	28.48
C. occidentalis	LM84	Claviceps	431	313	10.9	18.79
C. quebecensis	LM458	Claviceps	176	259	19.32	26.14
C. quebecensis	Clav32	Claviceps	189	284	14.29	24.34
C. quebecensis	Clav50	Claviceps	161	258	14.91	26.09
C. ripicola	LM218	Claviceps	386	523	16.84	31.61
C. ripicola	LM219	Claviceps	393	490	15.78	28.5
C. ripicola	LM220	Claviceps	412	412	16.02	28.64
C. ripicola	LM454	Claviceps	434	546	13.13	21.43
C. spartinae	CCC535	Claviceps	251	368	10.36	16.33
C. arundinis	CCC1102	Claviceps	431	518	11.6	22.51
C. arundinis	LM583	Claviceps	362	442	15.19	26.24
C. humidiphila	LM576	Claviceps	401	538	14.96	23.44
C. perihumidiphila	LM81	Claviceps	351	494	19.66	33.62
C. cyperi	CCC1219	Claviceps	193	244	5.7	7.77
C. pusilla	CCC602	Pusillae	9	17	0.0	0.0
C. fusiformis	PRL 1980	Pusillae	4	8	0.0	0.0
C. lovelessii	CCC647	Pusillae	7	14	0.0	0.0
C. digitariae	CCC659	Pusillae	10	18	0.0	0.0
C. maximensis	CCC398	Pusillae	3	6	0.0	0.0
C. sorghi	CCC632	Pusillae	12	23	0.0	8.33
C. africana	CCC489	Pusillae	8	16	0.0	0.0
C. citrina	CCC265	Citrinae	24	34	4.17	4.17
C. paspali	RRC 1481	Paspalorum	1	2	0.0	0.0

† Unique pairs (i.e. pairs of gene A : gene B and gene B : gene A are not counted twice).

Table S7: Means, standard deviations, and additional statistics of repeat-induced point mutation (RIP) composite indexes and large RIP affected regions (LRARs) for all 53 *Clvaiceps* genomes computed using The RIPper on default settings.

Species	Strain	Section	RIP composite index†	RIP affected windows (#)	RIP genomic content (%)	LRARs (#)	LRARs length (kbp)	LRARs genomic content (kbp)	LRARs composite index†	LRARs GC content (%)
C. purpurea	20.1	Claviceps	-0.61 ± 0.29	80	0.12%					
C. purpurea	Clav04	Claviceps	-0.59 ± 0.28	136	0.21%					
C. purpurea	Clav26	Claviceps	-0.59 ± 0.28	86	0.14%					
C. purpurea	Clav46	Claviceps	-0.59 ± 0.28	88	0.14%					
C. purpurea	Clav55	Claviceps	-0.59 ± 0.28	96	0.15%					
C. purpurea	LM4	Claviceps	-0.59 ± 0.28	75	0.12%					
C. purpurea	LM5	Claviceps	-0.59 ± 0.28	75	0.12%					
C. purpurea	LM14	Claviceps	-0.59 ± 0.28	66	0.11%					
C. purpurea	LM28	Claviceps	-0.59 ± 0.28	79	0.13%					
C. purpurea	LM30	Claviceps	-0.59 ± 0.28	71	0.12%					
C. purpurea	LM33	Claviceps	-0.59 ± 0.28	76	0.12%					
C. purpurea	LM39	Claviceps	-0.59 ± 0.28	68	0.11%					
C. purpurea	LM46	Claviceps	-0.59 ± 0.28	84	0.14%					
C. purpurea	LM60	Claviceps	-0.59 ± 0.28	61	0.1%					
C. purpurea	LM71	Claviceps	-0.59 ± 0.28	79	0.13%					
C. purpurea	LM207	Claviceps	-0.59 ± 0.28	78	0.13%					
C. purpurea	LM223	Claviceps	-0.59 ± 0.28	94	0.15%					
C. purpurea	LM232	Claviceps	-0.59 ± 0.28	64	0.1%					
C. purpurea	LM233	Claviceps	-0.59 ± 0.28	73	0.12%					
C. purpurea	LM461	Claviceps	-0.59 ± 0.28	72	0.12%					
C. purpurea	LM469	Claviceps	-0.59 ± 0.28	76	0.12%					
C. purpurea	LM470	Claviceps	-0.59 ± 0.28	90	0.15%					
C. purpurea	LM474	Claviceps	-0.59 ± 0.28	94	0.15%					
C. purpurea	LM582	Claviceps	-0.59 ± 0.28	81	0.13%					
C. aff. purpurea	Clav52	Claviceps	-0.59 ± 0.31	65	0.11%					
C. capensis	CCC1504	Claviceps	-0.61 ± 0.27	41	0.07%					
C. pazoutovae	CCC1485	Claviceps	-0.61 ± 0.27	39	0.07%					
C. monticola	CCC1483	Claviceps	-0.59 ± 0.28	45	0.08%					
C. occidentalis	LM78	Claviceps	-0.55 ± 0.31	119	0.2%					
C. occidentalis	LM77	Claviceps	-0.55 ± 0.31	133	0.23%					
C. occidentalis	LM84	Claviceps	-0.55 ± 0.31	111	0.19%					
C. quebecensis	LM458	Claviceps	-0.57 ± 0.29	88	0.15%					
C. quebecensis	Clav32	Claviceps	-0.57 ± 0.29	83	0.14%	2	4.5 ± 0.01	9.0	0.73 ± 0.05	54.07% ± 0.37%
C. quebecensis	Clav50	Claviceps	-0.57 ± 0.29	76	0.13%					
C. ripicola	LM218	Claviceps	-0.57 ± 0.29	82	0.13%					
C. ripicola	LM219	Claviceps	-0.57 ± 0.28	87	0.14%					
C. ripicola	LM220	Claviceps	-0.58 ± 0.29	85	0.14%					
C. ripicola	LM454	Claviceps	-0.57 ± 0.28	82	0.13%					
C. spartinae	CCC535	Claviceps	-0.58 ± 0.28	73	0.12%					
C. arundinis	CCC1102	Claviceps	-0.58 ± 0.28	55	0.09%					
C. arundinis	LM583	Claviceps	-0.58 ± 0.28	60	0.1%					
C. humidiphila	LM576	Claviceps	-0.59 ± 0.28	64	0.1%					
C. perihumidiphila	LM81	Claviceps	-0.58 ± 0.28	80	0.13%					
C. cyperi	CCC1219	Claviceps	-0.60 ± 0.27	90	0.17%	1	4.5 ± 0	4.5	0.66 ± 0.00	53.18% ± 0.00%
C. pusilla	CCC602	Pusillae	0.15 ± 1.04	36,205	38.36%	564	13.7 ±11.2	7,739	1.30 ± 0.17	25.23% ± 4.05%
C. fusiformis	PRL 1980	Pusillae	0.03 ± 1.30	18,107	16.67%	274	5.9 ± 1.6	1,610	1.84 ± 0.47	4.81% ± 3.15%
C. lovelessii	CCC647	Pusillae	-0.02 ± 1.00	25,695	30.29%	399	10.8 ± 6.6	4,320	1.32 ± 0.16	23.55% ± 3.80%
C. digitariae	CCC659	Pusillae	-0.25 ± 0.89	13,661	20.17%	271	11.5 ± 7.5	3,109	1.37 ± 0.15	22.13% ± 3.38%
C. maximensis	CCC398	Pusillae	-0.24 ± 0.91	13,517	20.37%	148	14.6 ± 13.5	2,156	1.43 ± 0.13	21.44% ± 2.49%
C. sorghi	CCC632	Pusillae	0.01 ± 1.04	21,622	29.57%	348	13.8 ± 13.4	4,804	1.41 ± 0.16	23.68% ± 2.79%
C. africana	CCC489	Pusillae	0.04 ± 0.99	25,266	33.09%	289	22.0 ± 19.7	6,362	1.33 ± 0.09	20.11% ± 3.85%
C. citrina	CCC265	Citrinae	0.36 ± 1.11	43,520	48.69%	503	9.9 ± 6.4	4,957	1.34 ± 0.17	29.80% ± 3.95%
C. paspali	RRC 1481	Paspalorum	-0.35 ± 1.00	5,351	9.05%	131	6.1 ± 1.6	799	1.85 ± 0.39	4.11% ± 2.75%

† *Composite Index Value* [(TpA/ ApT) – (CpA + TpG/ ApC + GpT)], positive values imply RIP
